# Host evolution shapes gut microbiome composition in *Astyanax mexicanus*

**DOI:** 10.1002/ece3.11192

**Published:** 2024-04-01

**Authors:** Misty R. Riddle, Nguyen K. Nguyen, Maeve Nave, Robert Peuß, Ernesto Maldonado, Nicolas Rohner, Clifford J. Tabin

**Affiliations:** ^1^ University of Nevada, Reno Reno Nevada USA; ^2^ Microbiome Insights Inc Vancouver British Columbia Canada; ^3^ Institute for Evolution and Biodiversity University of Münster Münster Germany; ^4^ Institute of Marine Sciences and Limnology Universidad Nacional Autonoma de Mexico, UNAM Puerto Morelos Mexico; ^5^ Stowers Institute for Medical Research Kansas City Missouri USA; ^6^ Harvard Medical School Blavatnik Institute Boston Massachusetts USA

**Keywords:** *Astyanax mexicanus*, cavefish, *Cetobacterium*, evolution, Fusobacteriota, gut, microbiome, phylosymbiosis

## Abstract

The ecological and genetic changes that underlie the evolution of host–microbe interactions remain elusive, primarily due to challenges in disentangling the variables that alter microbiome composition. To understand the impact of host habitat, host genetics, and evolutionary history on microbial community structure, we examined gut microbiomes of river‐ and three cave‐adapted morphotypes of the Mexican tetra, *Astyanax mexicanus*, in their natural environments and under controlled laboratory conditions. Field‐collected samples were dominated by very few taxa and showed considerable interindividual variation. We found that lab‐reared fish exhibited increased microbiome richness and distinct composition compared to their wild counterparts, underscoring the significant influence of habitat. Most notably, however, we found that morphotypes reared on the same diet throughout life developed distinct microbiomes suggesting that genetic loci resulting from cavefish evolution shape microbiome composition. We observed stable differences in Fusobacteriota abundance between morphotypes and demonstrated that this could be used as a trait for quantitative trait loci mapping to uncover the genetic basis of microbial community structure.

## Graphical abstract

The ecological and genetic changes that underlie the evolution of host–microbe interactions remain elusive. We examined gut microbiomes of river‐ and three cave‐adapted morphotypes of the Mexican tetra, *Astyanax mexicanus*, in their natural environments and under controlled laboratory conditions to reveal how host habitat, host genetics, and evolutionary history of microbial community structure.

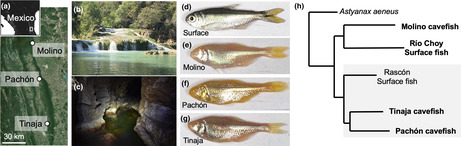

## 1 INTRODUCTION

The gastrointestinal (GI) microbiome plays a critical role in host fitness by facilitating nutrient absorption and producing metabolites that influence interaction with the ecological niche (Greene et al., 2020), tissue homeostasis (Bates et al., 2006), the immune system (Wang et al., 2021), and behavior (Gulledge et al., 2023). The host–microbe relationship begins during development and changes throughout life depending on environmental variables like diet (Carmody et al., 2015; David et al., 2014; Leigh et al., 2022; Staubach et al., 2013). Host‐driven mechanisms that shape microbiome composition may provide individuals with a selective advantage (Ganal‐Vonarburg et al., 2020; Gould et al., 2018; Jia et al., 2021). A study comparing various species of adult deer mice, mosquitos, wasps, and great apes showed that variation in the microbiome is less within species than between species, and as species diverge, microbial communities become more distinct (Brooks et al., 2016). This phenomenon, known as phylosymbiosis, provides evidence that microbiome composition is associated with host evolution (Lim & Bordenstein, 2020). However, the host genetic changes that underlie natural variation in microbiome composition are largely unknown and challenging to investigate in most systems. Such studies require comparing the microbiomes of closely related genetically tractable species or populations that have adapted to known environments. Studying divergent populations of the same species thrust into similar conditions would allow investigation of parallel development of host genetic mechanisms that select microbial communities.

**FIGURE 1:**
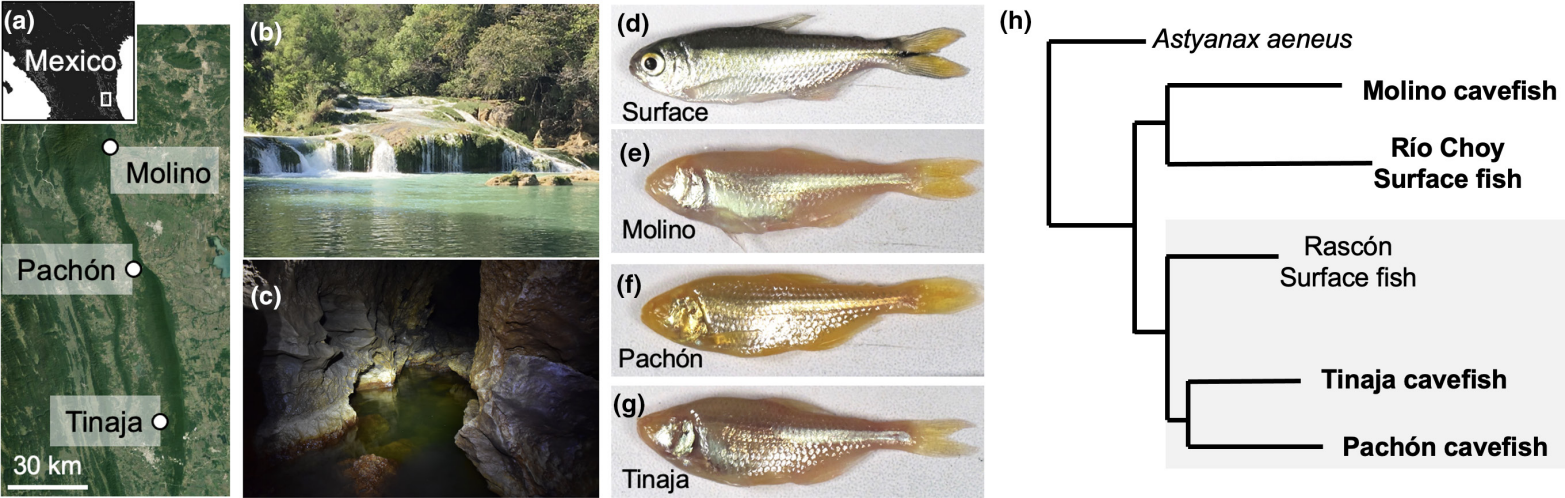
Geographic distribution, habitat, appearance and phylogeny of *Astyanax mexicanus*. (a) Map and sattelite image showing location of *A. mexicanus* cave morphotype populations used in this study. (b) Image of river in Mexico where *A. mexicanus* surface fish morphotypes are found. (c) Image of Pachón Cave where *A. mexicanus* cavefish morphotypes are found. (d–g) Images of surface fish and cavefish (Molino, Pachón, Tinaja) raised in the laboraotry. (h) *A. mexicanus* phylogeny; Tinaja and Pachón form a monophyletic clade referred to as the “old lineage” (gray shaded area) and Molino and Río Choy surface fish form a monophyletic clade referred to as the “new lineage.” Adapted from Ponnimbaduge Perera et al. (2023) with permission. Drawing based on Herman et al. (2018).

In this study, we use river and cave‐adapted populations of the Mexican tetra, *Astyanax mexicanus*, to investigate how host evolution shapes intestinal microbiome composition. *A. mexicanus* is a single species of fish consisting of river‐adapted surface fish and multiple eyeless cave‐adapted cavefish populations named for the dark limestone caves they inhabit in Northeastern Mexico (e.g., Tinaja, Molino, Pachón, Figure 1a–g). Cavefish have adapted to an environment with no light, reduced temperature variability (Tabin et al., 2018), low oxygen (Ornelas‐García et al., 2018), limited nutrients (Espinasa et al., 2017; Wilson et al., 2021), reduced competition (Elliott, 2016), and altered parasite diversity (Peuß et al., 2020; Santacruz et al., 2023). Cavefish populations exhibit similar changes in their morphology, like eye loss and reduced pigmentation (Krishnan & Rohner, 2017; Protas et al., 2006), physiology, like starvation resistance and hyperphagia (Aspiras et al., 2015), and behavior, like loss of schooling and reduced sleep (Duboué et al., 2011; Kowalko, Rohner, Rompani, et al., 2013). Based on whole genome sequencing, the *A. mexicanus* phylogeny defines two lineages; Tinaja and Pachón cavefish form a monophyletic clade separate from Molino cavefish and Río Choy surface fish (Herman et al., 2018, Figure 1h). This evolutionary history indicates that cavefish traits may have evolved through repeated evolution.

Researchers have bred surface fish and cavefish in laboratories for generations to study the genetic and developmental basis of cavefish traits like reduced aggression (Rodriguez‐Morales et al., 2022), increased fat accumulation (Aspiras et al., 2015; Olsen et al., 2023; Xiong et al., 2022), and insulin resistance (Riddle, Aspiras, et al., 2018). These traits have been linked with microbiome composition in other species (Jia et al., 2021; Qin et al., 2012; Turnbaugh et al., 2006). Characterizing the microbiome of *A. mexicanus* therefore represents an important step in understanding cavefish evolution. A previous study that compared the stomach microbiome of two field‐collected Pachón cavefish and four field‐collected surface fish suggested that dissolved oxygen, more so than morphotype identity, determined microbial community composition (Ornelas‐García et al., 2018). Here, we characterize the intestinal microbiome of cavefish and surface fish that were collected in the field or raised in the laboratory to ask: (1) Do cavefish and surface fish in their natural habitat have different microbiomes? (2) How similar are the microbiomes of wild and laboratory‐raised *A. mexicanus*? (3) Does host evolutionary history shape microbiome composition in *A. mexicanus*? As anticipated, we found that the microbiome is impacted by habitat. Our data shows that laboratory‐raised fish harbor a more diverse microbiome that is significantly different in composition compared to their wild counterparts. More surprisingly, we found that host genetics alone can explain differences in microbiome composition between surface fish and cavefish. In addition, we found stable differences in the abundance of Fusobacteriota between laboratory‐raised populations suggesting that taxa abundance could be used as a quantitative trait for genetic mapping in *A. mexicanus* as has been done in mice (Kemis et al., 2019; Zhang et al., 2023). Our study defines the variables that shape the microbiome in a model system that will continue to impact our understanding of ecology and evolution due to its unique phylogeny (Herman et al., 2018; Ornelas‐García & Pedraza‐Lara, 2015), amenability to laboratory manipulation (Peuß et al., 2019; Riddle, Martineau, et al., 2018), and genetic accessibility (Klaassen et al., 2018; Stahl, Peuß, et al., 2019).

## 2 METHODS

### 2.1 Collection of intestinal contents from wild fish

The Pachón Cave is perpetually dark, and previous studies analyzing gut contents of field‐caught fish indicate that adults are opportunistic generalists with a seasonally variable diet of mostly detritus and bat guano (Espinasa et al., 2017; Wilson et al., 2021). In contrast, surface fish have a normal day/night cycle and consume insects, crustaceans, and annelids (Mills, 1989). Field collection of *Astyanax mexicanus* for this study was conducted under permit no. SGPA/DGVS/03634/19 granted by the Secretaría de Medio Ambiente y Recursos Naturales to Ernesto Maldonado. The date and time of collections, and measured water chemistry variables are reported in Table S1. Total intestinal contents were collected from wild Pachón cavefish and Río Choy surface fish in July 2019 using the following protocol: Captured fish were placed in their environmental water and euthanized by immersion in MS‐222 on the day of capture. The gut was removed by making a cut posterior to the stomach and directly at the urogenital pore. The gut was transferred to a sterile Petri dish and the contents were washed out by inserting a sterile syringe into one end and expelling 200 μL sterile PBS. The contents and PBS were transferred to 300 μL RNA later. The samples were stored at room temperature for 4 days, shipped on ice, and stored at −20°C until DNA extraction was carried out. The PBS and RNA later used to collect samples were included in the DNA extraction and sequencing pipeline and did not produce read counts high enough to analyze. Thirteen out of 20 wild samples that were collected had enough read counts to analyze: five Pachón (three males, one female, one undifferentiated) and six Río Choy (three males, three females). The weight of the fish used for sequencing is shown in Figure S1a.

### 2.2 Collection of intestinal contents from laboratory‐reared fish

Total intestinal contents were collected from Río Choy surface fish and Tinaja, Molino, and Pachón cavefish that were raised on the same recirculating water system and diet as described below. These fish originated from parents that were bred for at least five generations in the laboratory of Clifford Tabin at Harvard Medical School (TMR2, SMR1, PMR1, MMR7). To minimize the transfer of parental microbes to the embryos, we removed them from spawning tanks and incubated them in 0.3096% sodium hypochlorite (bleach) for 5 min, then 3.2 mM Sodium thiosulfate for 1 min, then rinsed them in fish‐ready water (adapted from Dahm & Nüsslein‐Volhard, 2002). The embryos were allowed to hatch in 1 L containers at a density of 20 fish per container. The fish were fed L‐type rotifers to 14 days post fertilization, then transferred to 20‐gallon tanks on the same recirculating system at a density of approximately 2 fish per liter of water. The fish were fed *Artemia fransiscana* until 60 days post fertilization, then New Life Spectrum Thera‐A Medium Sinking Pellets. At 6‐months post fertilization, fish were euthanized in MS‐222 5 h after eating. The fish were weighed, and total intestinal contents were collected using the same method used for field‐caught fish. Samples were stored at room temperature for 24 h, then stored at −20°C until DNA extraction was carried out. The sex of most surface fish samples was apparent while most cavefish samples did not have clearly visible gonads. Gut contents from nine surface (three male, four female, two undifferentiated), nine Pachón (one male, eight undifferentiated), eight Tinaja (eight undifferentiated), nine Molino (six male, three undifferentiated) were shipped on dry ice to Microbiome Insights Inc. (Vancouver, BC, Canada) for DNA isolation and sequencing. The weight and sex of fish used for sequencing is reported in Figure S1b and Table S2.

### 2.3 DNA extraction and 16S rRNA gene sequencing

Total intestinal contents from each fish were placed into a MoBio PowerMag Soil DNA Isolation Bead Plate. DNA was extracted following MoBio's instructions on a KingFisher robot. Bacterial 16S rRNA genes were PCR‐amplified with dual‐barcoded primers targeting the V4 region (515F 5′‐GTGCCAGCMGCCGCGGTAA‐3′, and 806R 5′‐GGACTACHVGGGTWTCTAAT‐3′), as per the protocol of Kozich et al. (2013). Amplicons were sequenced with an Illumina MiSeq using the 300‐bp paired‐end kit (v.3), with all samples of this study being included in the same run.

### 2.4 Data processing for microbiome analysis

Fastq files were imported and processed by Qiime2 (v 2021‐11) (Bolyen et al., 2019) [https://qiime2.org/] following the public tutorial for Casava 1.8 paired end demultiplexed sample format. Sequences were denoised using the DADA2 method (Callahan et al., 2016) via qiime data2 denoise‐paired to obtain an amplicon sequencing variant (ASV) table. Taxonomy was assigned to ASVs by qiime feature‐classifier (Bokulich et al., 2018) (classify‐sklearn) against the pre‐trained Silva v138 database (Quast et al., 2013) or V4 region (515F/806R) using RESCRIPt (Robeson et al., 2021). Untargeted sequences were removed. The potential for contamination was addressed by co‐sequencing DNA amplified from specimens and from two each of template‐free controls and extraction kit reagents processed the same way as the specimens. Control samples contained less than 34 read counts. Experimental samples with less than 1000 high‐quality read counts were excluded from downstream analysis. An average of 8197 quality‐filtered reads were generated per wild sample. An average of 23,408 quality‐filtered reads were generated per laboratory sample. The total number of ASVs from the wild and laboratory datasets was 4555 (including those occurring once with a count of 1, or singletons).

### 2.5 Quantification and statistical analysis

Alpha diversity was estimated by different indices including Shannon, Inverse Simpson, Observed Species, and Phylogenetic Index on raw count ASV table after filtering out contaminants using the Phyloseq R package (McMurdie & Holmes, 2013). The significance of diversity differences was tested with one‐way ANOVA with Tukey's post hoc test including morphotype and habitat as variables (Indices~morphotype*habitat). To estimate beta diversity across samples, we computed Bray‐Curtis, Unweighted Unifrac (Lozupone et al., 2011), and Jaccard distance using the vegan v2.6‐2 (Oksanen et al., 2015) and Phyloseq R package. We visualized differences across samples using Principal Coordinate Analysis (PCoA) ordination. Variation in microbial diversity was assessed with permutational multivariate analyses of variance (adonis in R) with population and location as factors using 9999 permutations for significance testing. To generate the microbiota dendrogram we collapsed raw ASV table counts from laboratory samples by host morphotype identity to get representative microbiota profiles using the microbiome R package (Lahti & Shetty, 2017). We calculated unweighted unifrac distances for each table and the matrices were Unweighted Pair Group Method with Arithmetic mean (UPGMA) clustered to generate dendrograms of interspecific relatedness using phangorn v 2.9.0 (Schliep, 2011) and ggtree (Yu, 2020) packages. All analyses were conducted in the R environment (R‐Core‐Team, 2019). Data were plotted using the ggplot2 R package (Wickham, 2016).

### 2.6 Quantitative PCR of Fusobacteriota abundance

Siblings of the surface fish and Pachón cavefish used for microbiome sequencing (SMR1, PMR1) were shipped from Harvard Medical School to University of Nevada, Reno, in March 2021 when they were 20 months old. Fish were housed in 9 L tanks with 10 fish per tank under 6500 K daylight LEDs and fed ⅛ tsp of New Life Spectrum Thera+A Regular Pellet Enhanced Non‐Medicated Fish Food per day for 4 months before fecal collections. To collect feces, individual fish were moved to 2 L tanks and fed five pellets of New Life Spectra Thera A+ per day. On the third day of isolation, tanks were cleared of any debris, the fish were fed, and the inlet water was turned off. Feces were collected from the tank after 24 h. The water inlet was turned on to recirculate fresh water and the process was repeated over 3 days. Fecal samples were centrifuged, excess water was removed, and they were stored at −80°C until DNA extraction. DNA was extracted from the fecal samples using Zymo Quick‐DNA Fecal/Soil Microbe Microprep (D6012). DNA concentration was quantified using an Invitrogen Qubit 4 Fluorometer with the 1× dsDNA High Sensitivity assay kit (Q33230). Quantitative PCR was performed on a BioRad CFX96 Touch Real‐Time PCR machine using the following primers to amplify 16s rRNA genes from Fusobacteriota (Fwd: 5′‐GGATTTATTGGGCGTAAAGC‐3′; Rev: 5′‐GGCATTCCTACAAATATCTACGAA‐3′) (Boutaga et al., 2005). We also included reactions to amplify 16s rRNA genes from all Eubacteria to normalize the results to the total amount of bacterial DNA (Fwd 5′‐GGTGAATACGTTCCCGG‐3′; Rev 5′‐TACGGCTACCTTGTTACGACTT‐3′) (Kostic et al., 2013). Each reaction contained 1× iTaq™ Universal SYBR® Green Supermix, 100 nM of each primer, and 1.5 ng of DNA. The cycle conditions were 95°C for 5 min, followed by 40 cycles of 95°C for 5 s and 57°C for 30 s. Melt curve analysis was performed at 65–95°C with 5°C increments 5 s/step. Reactions without fecal DNA showed amplification of Eubacterial sequences at high cycle number (>29) consistent with the manufacturing of Taq DNA polymerase (Corless et al., 2000). Fusobacteriota was not detected in samples without fecal DNA.

## 3 RESULTS

### 3.1 Intestinal microbiome of *A. mexicanus* cavefish and surface fish in their natural habitats

We first analyzed the microbiome of *A. mexicanus* surface fish and cavefish morphotypes by comparing fish that were captured in the Rio Choy River and Pachón Cave, respectively. In addition to consuming distinct diets, the fish reside in water with dissimilar chemical qualities; water in the Pachón cave at the time of collection was lower in temperature, dissolved oxygen, conductance, copper, and phosphate (Table S1). Despite these distinct habitats, we did not observe significant differences in the richness or composition of the intestinal microbiome between fish collected at each site (Shannon Index one‐way ANOVA with Tukey's post hoc test *p*.adj = .84, Beta diversity Permanova with pairwise post hoc test *p*.adj = .098).

**FIGURE 2:**
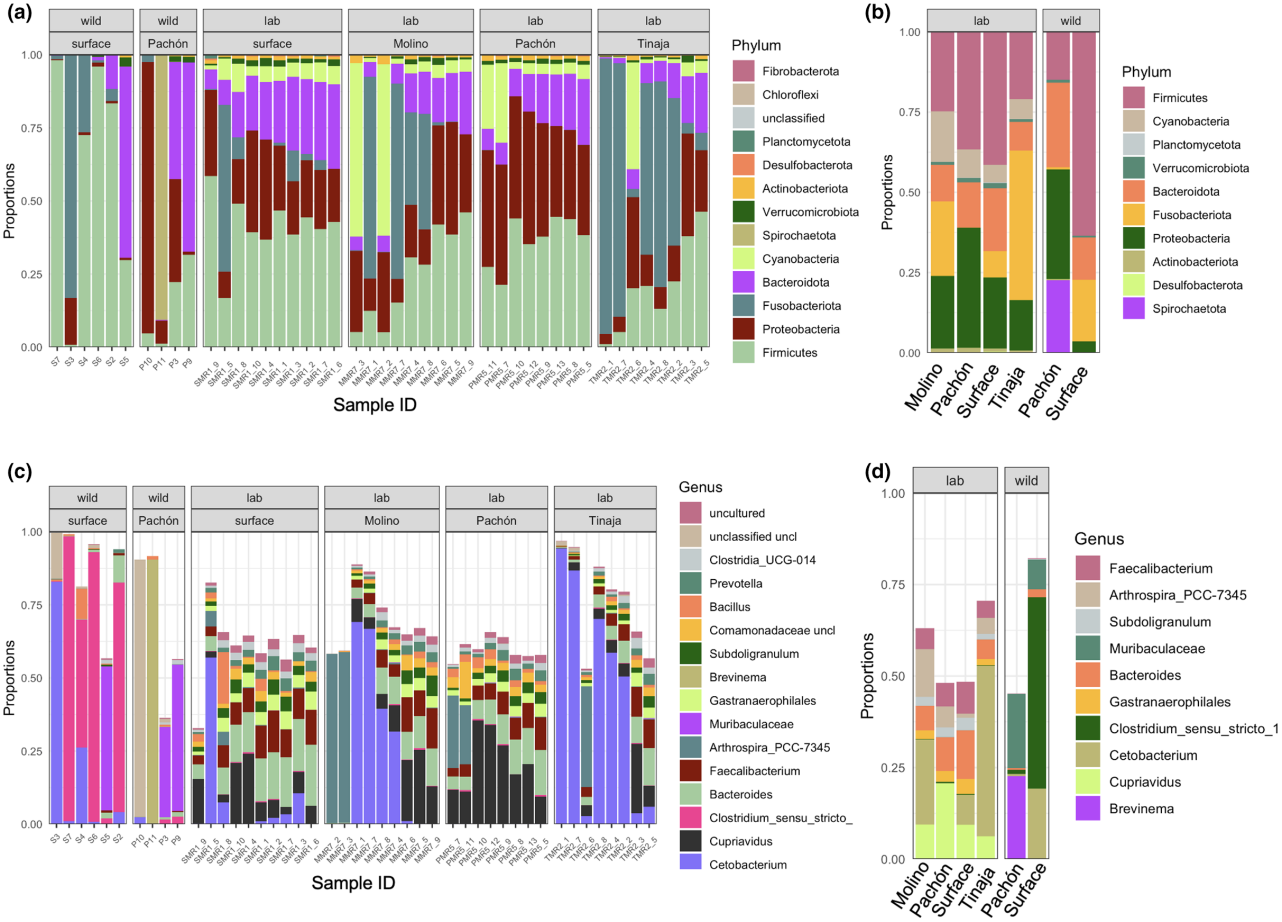
Taxonomic composition of the intestinal microbiome of wild and laboratory‐raised *Astyanax mexicanus* surface fish and cavefish morphotypes. (a) Proportional abundance by phylum of ASVs identified in wild samples (surface and Pachón) and laboratory raised samples (surface, Pachón, Tinaja, and Molino). (b) Average proportional abundance by phylum of ASVs identified in wild samples and laboratory raised samples. (c) Proportional abundance by genus of ASVs identified in wild samples and laboratory raised samples. (d) Average proportional abundance by genus of ASVs identified in wild samples and laboratory raised samples.

We examined the proportional abundance of taxa and found that many fish were dominated by only one or two phyla, and although there was a considerable interindividual variation comparing samples, some phyla that were consistently abundant in surface fish were at low abundance in Pachón cavefish (Figure 2). For example, we observed that Firmicutes were the most abundant phylum in four out of six surface fish and, in contrast, were at relatively low abundance in all four Pachón cavefish samples. Pachón individuals instead were dominated by Proteobacteria, Bacteroidota, or Spirochaetota (Figure 2a,b, average proportional abundance (APA) of Firmicutes = 0.15 Pachón, 0.64 surface, Proteobacteria = 0.62 Pachón, 0.03 surface, Bacteroidota = 0.12 Pachón, 0.09 surface). Similarly, four out of six surface fish samples were dominated by the genus *Clostridium*, which was found at very low abundance in only two out of four Pachón cavefish samples (Figure 2c, APA = 0.008 Pachón, 0.52 surface). In addition, two surface fish samples (s3, s4) had a high proportion of the genus *Cetobacterium* which was found at very low abundance in only one Pachón cavefish (Figure 2c, APA = 0.07 Pachón, 0.20 surface). In contrast, the genus *Brevinema* was observed at high abundance in one of the Pachón cavefish and was not found in surface fish. *Brevinema* was first characterized as an infectious pathogen in rodents (Anderson et al., 1987). In fish, abundance has been associated with high stocking density and upregulation of immune gene expression (Brown et al., 2019; Li et al., 2021; Tapia‐Paniagua et al., 2014), suggesting it could represent a pathogen.

We considered that interindividual variation in phyla abundance could be driven by fish weight; although the average weight was not different between populations, four of the field‐collected surface fish (S2, S3, S4, S5) were considerably larger than cavefish (Table S2, Figure S1, *p* = .08, *t*‐test). However, we found that the abundance of Bacteriodota, for example, did not correlate with fish weight (*p* > .05, Spearman's). Similarly, we did not find a significant effect of fish weight for any of the alpha diversity measures (*p*‐values for Shannon: .1464, InvSimpson: .592, observed diversity .2751, Phylogenetic diversity .1788). Overall, the current data suggests that in their natural habitat, *A. mexicanus* surface fish and cavefish gut microbiomes are dominated by only a few phyla and that some phyla that are found at high abundance in surface fish are found at low abundance in Pachón cavefish. Since a limited number of samples are available for field collection, it is not possible to control for the age or size of the fish to determine how these variables impact microbiome composition. To address this limitation, we next compared the microbiome of fish bred and reared in the laboratory.

### 3.2 Comparison of the intestinal microbiome between wild and laboratory‐reared *A. mexicanus*

**FIGURE 3:**
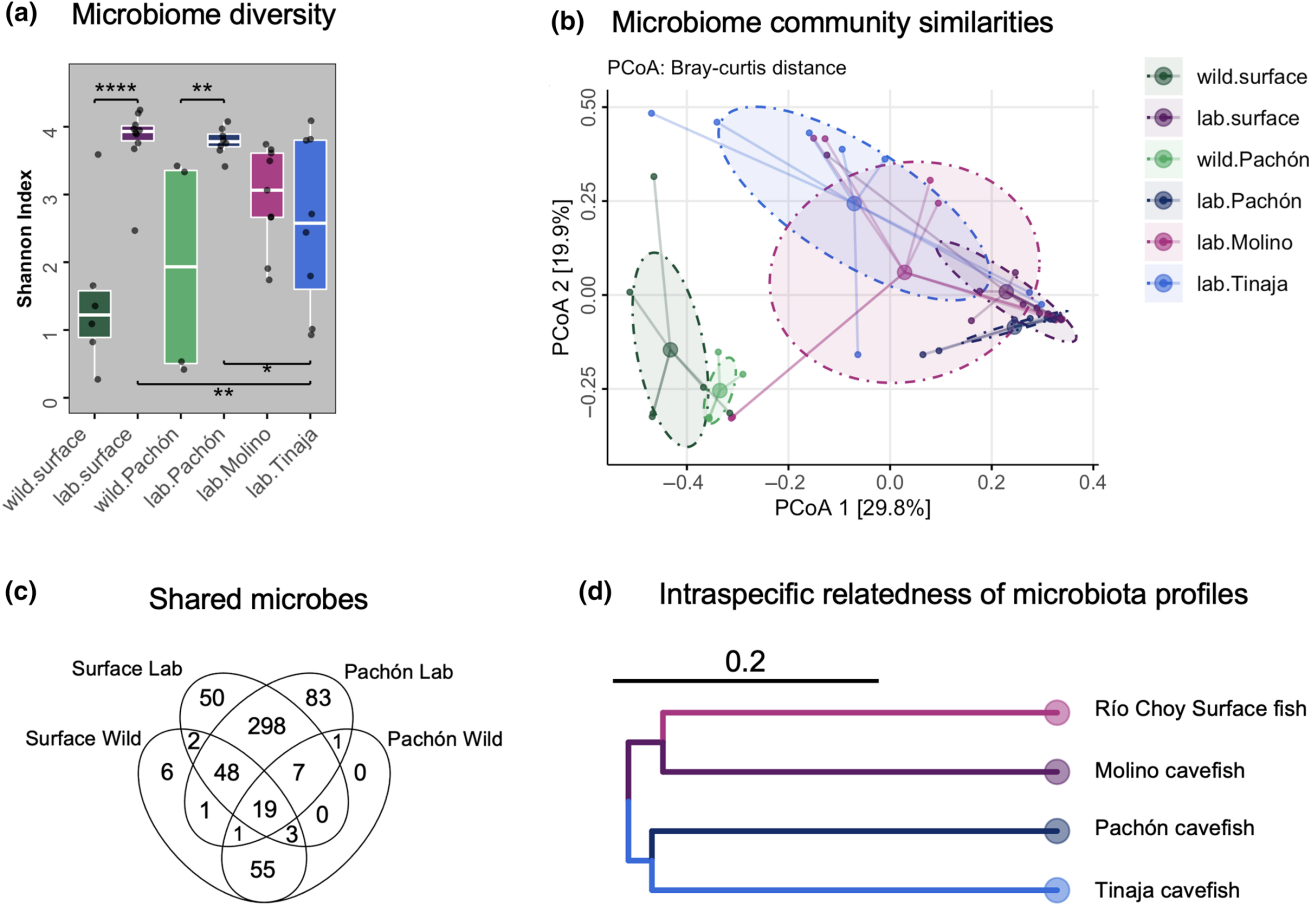
Comparison of the gut microbiome between wild and laboratory‐raised *Astyanax mexicanus* surface fish and cavefish morphotypes. (a) Comparison of the number of species and their distributions in intestinal microbiome of *A. mexicanus* surface and cavefish populations as estimated by Shannon Index. Asterisks indicate significance based on one‐way ANOVA with Tukey's post hoc test (*p < .05, **p < .005). (b) Variation in intestinal microbiome composition between *A. mexicanus* surface fish and cavefish in the wild and lab. Distances determined by Bray‐Curtis. Ellipses estimate covariance and the centroid of each cluster. PCoA used as the ordination method. (c) Venn diagram showing shared number of ASVs between wild and laboratory‐raised surface fish and Pachón cavefish. (d) Dendrogram of interspecific relatedness of the intestinal microbiome of laboratory‐raised *A. mexicanus*. ASV tables were collapsed by the indicated population and Bray‐Curtis distances were calculated for each representative microbiota profile. The resulting Bray‐Curtis distance matrices were UPGMA clustered to produce the dendrogram.

To understand how similar the microbiome of *A. mexicanus* in their wild habitat is to those raised in the laboratory, we compared the microbiomes of field‐collected surface fish and Pachón cavefish to their laboratory‐reared counterparts that had been bred in the laboratory for generations and were raised on the same diet and in the same water. The laboratory habitat is different from both the cave and river habitats. In the laboratory, the fish experience lower temperature and pH, higher conductance and dissolved oxygen, an invariable light cycle, more crowded growth conditions, and a consistent high‐nutrient diet (Table S1). We found that laboratory‐raised fish had the greater richness of microbial species in the intestinal microbiome compared to fish in the wild habitat (Figure 3a, Shannon Index, Figure S2, total observed species, phylogenetic index); alpha‐diversity was significantly different in samples from surface fish and Pachón cavefish collected in the field compared to surface fish and Pachón cavefish raised in the lab (Shannon Diversity Tukey's post‐hoc test, wild surface vs. lab surface FDR *p*.adj = .001, wild Pachón vs. lab Pachón FDR *p*.adj = .008, Table S3). Our results suggest the laboratory habitat supports the growth of a richer microbial community in the GI tract than in either natural environment.

To obtain a graphical representation of the differences in microbiome composition between the wild and the laboratory‐reared fish we summarized ASV abundances into Bray‐Curtis dissimilarities and performed principle coordination analysis (PCoA, Figure 3b). In the PCoA plot, wild surface fish and wild Pachón cavefish form two partially overlapping clusters that are separate from laboratory‐raised fish. We found that habitat (wild vs. lab) significantly impacted microbiome composition (Table 1, *adonis* R function, or Permanova, *p* = .002); beta‐diversity was significantly different in samples from surface fish and Pachón cavefish collected in the wild compared to surface fish and Pachón cavefish raised in the lab (Table 2, pairwise post‐hoc test, wild surface vs. lab surface FDR *p*.adj = .005, wild Pachón vs. lab Pachón FDR *p*.adj = .006). In summary, the intestinal microbiome of *A. mexicanus* in the laboratory has increased richness and altered composition compared to *A. mexicanus* in their natural habitat. These results have implications for researchers investigating the evolution of cavefish traits that may be influenced by microbiome composition.

**TABLE 1:** Statistical comparison of microbiota composition in *A. mexicanus* gut contents using results of 16s rRNA gene sequencing.

	*R* ^2^	Pr(>F)
Habitat	.2152	.001
Morphotype	.1292	.002
Habitat: Morphotype	.0401	.016
Residuals	.6155	
Total	1	

*Note*: Permutational analysis of variance test (adonis R function) determined significant differences in beta‐diversity among habitats (lab vs. wild), *A. mexicanus* morphotypes (surface, Pachón, Tinaja, Molino), and morphotype: habitat factors. *R* ^2^ represents the proportion of the variance explained by the independent variable(s). Pr(>F) represents the *p*‐value associated with an *F*‐statistic.

**TABLE 2:** Statistical comparison of microbiota composition in *A. mexicanus* gut contents using results of 16s rRNA gene sequencing.

Pairs	*R* ^2^	*p*.adjusted
Wild surface vs. wild Pachón	.212	.098
Wild surface vs. lab surface	.43	.005
Wild surface vs. lab Pachón	.462	.005
Wild surface vs. lab Tinaja	.325	.005
Wild surface vs. lab Molino	.281	.006
Wild Pachón vs. lab surface	.39	.006
Wild Pachón vs. lab Pachón	.411	.006
Wild Pachón vs. lab Tinaja	.327	.006
Wild Pachón vs. lab Molino	.256	.013
Lab surface vs. lab Pachón	.14	.025
Lab surface vs. lab Tinaja	.238	.02
Lab surface vs. lab Molino	.104	.075
Lab Pachón vs. lab Tinaja	.301	.006
Lab Pachón vs. lab Molino	.122	.131
Lab Tinaja vs. lab Molino	.065	.387

*Note*: Post‐hoc pairwise test with Shannon reveals difference in intestinal microbiome beta‐diversity between *A. mexicanus* morphotypes. FDR method was used to correct *p*‐values for multiple comparisons (*p*.adjusted). *R* ^2^ represents the proportion of the variance explained by the independent variable(s).

### 3.3 Shared microbial taxa between wild and laboratory‐reared *A. mexicanus*

We next asked if any of the microbes present in the natural habitat have been maintained in the laboratory‐reared fish by identifying ASVs that are present in both sample types. We found that 53% of the microbes in the wild surface fish were present in the lab surface fish (72/135 amplicon sequencing variant (ASVs), Figure 3c). In comparison, 33% of the microbes in the wild Pachón cavefish were present in the lab Pachón cavefish (28/86 ASVs, Figure 3c). Microbes that are shared between individuals of the same species that have been raised in different habitats have been referred to as the “core microbiome.” It is hypothesized that these microbes are essential for the normal function of the host and that host‐driven mechanisms may exist to ensure they are represented (Roeselers et al., 2011). By this definition, Pachón cavefish had fewer core microbes compared to surface fish (28 vs. 72 ASVs), and most of the core microbes were also present in the surface fish core microbiome (19 out of the 28 ASVs). The data may suggest that many of the same microbes are essential for host function in surface fish and cavefish, but cavefish could require fewer. Exploring this possibility would require manipulating microbiome composition and studying the impact on the host. The core microbes are mostly from the phylum Firmicutes (Figure S4). Firmicutes are one of the dominant taxa in fish microbiomes and are important for carbohydrate metabolism (Kim et al., 2021). We only identified one microbe that was part of the Pachón core and never found in surface fish, *Aeromonas* asv.13 (Figure 3c, Figure S4). Aeromonas in fish can exhibit either pathogenic or mutualistic interactions, contingent upon the specific species and host involved (Bates et al., 2006; Li et al., 2020; Rolig et al., 2018; Stephens et al., 2016). Determining whether this “core microbe” reflects a difference in predisposition to infection, rather than a difference in maintenance of microbes that are essential to the host, will require species‐level identification and additional experiments. Overall, our results suggest that *A. mexicanus* in the laboratory has a microbiome that is distinct and more diverse compared to their wild counterparts. In addition, more microbes are shared between lab and wild surface fish compared to lab and wild cavefish.

### 3.4 Evidence that host identity drives differences in *A. mexicanus* microbiome composition

We next tested whether the identity of the host population impacts microbiome composition independent of habitat by comparing the intestinal microbiome of 6‐month‐old fish from the Río Choy River, Pachón Cave, Tinaja Cave, and Molino Cave that have been bred in the laboratory for generations, were surface sterilized as embryos, and were raised on the same recirculating water system and diet. In this controlled setting, we found that Tinaja and Molino cavefish had significantly lower microbial species richness compared to surface fish suggesting that genetic differences between hosts that shape host traits impact alpha diversity, the number and distribution of microbial species in the intestine (Figure 3a: Shannon Index, Figure S2b: Inverse Simpson Index).

In addition to impacting microbial species richness, we found that morphotype identity had a significant impact on microbiome composition (Table 1, Beta Diversity Permanova, *p* = .002). To visualize the differences in microbiome composition we summarized ASV abundances into Bray‐Curtis dissimilarities and performed principle coordination analysis (PCoA, Figure 3b). In the PCoA plot comparing laboratory raised fish, the centroid of Tinaja cavefish samples is furthest from surface fish samples and the ellipses estimating covariance overlap except for Pachón and Tinaja cavefish samples (Figure 3b). PCoA plots of unweighted unifrac and Jaccard distances differently show that the Pachón centroid is ordinated furthest from surface fish and that ellipses estimating covariances overlap in all samples (Figure S3). To determine which laboratory‐reared morphotypes harbored significantly different microbiomes, we performed a pairwise post‐hoc test (Table 2). We found that the microbiome of surface fish was significantly different from Pachón and Tinaja cavefish (*p*.adj = .025 and .020) but not Molino cavefish (*p*.adj = .075). These results suggest that, through the course of evolution, Tinaja and Pachón cavefish have acquired genetic changes that result in the establishment of a different microbiome from surface fish despite being raised in the same habitat. We also found that samples from Pachón and Tinaja cavefish were significantly different from each other suggesting distinct cavefish populations have genetic differences that impact microbiome composition (*p*.adj = .006). Since the fish were all raised under identical conditions, these results suggest that genetic and phenotypic differences between *A. mexicanus* morphotypes drive differences in microbiome composition.

### 3.5 Differential abundance of microbial taxa in laboratory‐reared *A. mexicanus*

We next compared the microbial taxa between laboratory‐raised *A. mexicanus* morphotypes to gain insight into the potential functional significance of host‐driven microbiome differences. We found that the four most abundant phyla were Firmicutes, Proteobacteria, Fusobacteriota, and Bacteroidota like in other freshwater fishes (Kim et al., 2021) (Figure 2a,b, Table S4). However, the average proportional abundance of Firmicutes was highest in surface fish compared to cavefish which recapitulates what we observed in the natural habitat (Figure 2a,b, APA = 0.40 surface, 0.36 Pachón, 0.21 Tinaja, 0.24 Molino). In addition, the phylum Bacteroidota and genus *Bacteroides* made up a high proportion of ASVs in surface fish samples compared to cavefish samples (Figure 2c,d, APA = 0.14 surface, 0.10 Pachón, 0.06 Tinaja, 0.07 Molino). We found that Pachón cavefish samples were mostly dominated by Proteobacteria of the genus *Cupriavidus* which was lower in the other morphotypes (Figure 2c,d, APA = 0.19 Pachón, 0.08 surface, 0.06 Tinaja, 0.09 Molino).

The most striking difference in taxonomic composition we observed was a very low abundance of Fusobacteriota in Pachón cavefish compared to the other morphotypes (Figure 2a: dark green bars, Figure 2b: light orange bars). We found that the Fusobacteriota phylum was consistently present in surface fish and was the most abundant phylum in 1 out of 10 surface fish. Proportional abundance of Fusobacteriota was greater in Molino and Tinaja cavefish samples compared to surface fish samples; it was the most common phylum in four out of nine Molino samples and five out of nine Tinaja samples. In contrast, we did not observe it in most Pachón samples (six out of eight) or it was at very low abundance if present (APA = 0.0005 Pachón, 0.08 surface, 0.46 Tinaja, 0.22 Molino). We found that samples from wild Pachón cavefish also had a low proportional abundance of Fusobacteriota compared to samples from wild surface fish (APA = 0.07 Pachón, 0.20 surface). The Fusobacteriota sequences we identified in the *A. mexicanus* gut microbiome are assigned to the genus *Cetobacterium* which is common in the microbiome of freshwater fish (Tsuchiya et al., 2007). Our data suggest that genetic differences between *A. mexicanus* morphotypes alter the taxonomic composition of the intestinal microbiome and that most strikingly, Pachón cavefish have dramatically reduced abundance of *Cetobacterium*.

### 3.6 Evidence of phylosymbiosis in *A. mexicanus*

We next tested if the differences in microbiome composition we observed between laboratory‐reared surface fish and cavefish mirror the evolutionary history of *A. mexicanus*. The *A. mexicanus* phylogeny defines two lineages; fish from the Pachón and Tinaja caves and Rascón River form a monophyletic clade referred to as the “old lineage” and fish from the Molino Cave and Río Choy River form a monophyletic clade referred to as the “new lineage” (Figure 1h). Based on whole genome sequencing data, old and new lineages split as long as 257 K generations ago with instances of secondary contact (Herman et al., 2018). Molino cavefish split from Río Choy Surface fish around 163 k generations ago, and Pachón and Tinaja cavefish split more recently around 116 k generations ago (Herman et al., 2018). We generated dendrograms of interspecific relatedness using the microbiota profiles of laboratory‐reared Pachón, Tinaja, and Molino cavefish and Río Choy surface fish. We found that the topology of the microbiota tree matches the topology of the host phylogeny (Figure 3d). The trees similarly define two lineages; Molino cavefish and Río Choy surface fish form a monophyletic clade that is separate from Pachón and Tinaja cavefish. Our results suggest that phylosymbiosis can occur in the same species that consists of distinct populations that diverged less than 300 K generations ago and adapted to dramatically different habitats.

### 3.7 Feasibility of genetically mapping microbiome composition in *A. mexicanus*

Despite their distinct appearance and behavior, *A. mexicanus* surface and cave morphotypes have remained interfertile. F2 surface/cave hybrids have been used in numerous quantitative trait loci (QTL) mapping studies to identify genetic changes associated with cavefish evolution (Gross et al., 2014; Klaassen et al., 2018; Kowalko, Rohner, Linden, et al., 2013; Protas et al., 2007; Riddle et al., 2021; Warren et al., 2021). Using microbiome composition as a trait for QTL mapping in mice has revealed genomic regions associated with the abundance of bacterial taxa in the intestine (Zhang et al., 2023). We next investigated the feasibility of using proportional abundance of taxa in the microbiome as a trait for QTL mapping in *A. mexicanus* by examining how stable bacterial taxa abundance is across time in individual fish. We reasoned that if the trait is highly plastic, it may be less likely to identify genetic markers associated with trait variance at the population level. We focused on Fusobacteriota since proportional abundance of this taxa was the most different between morphotypes.

**FIGURE 4:**
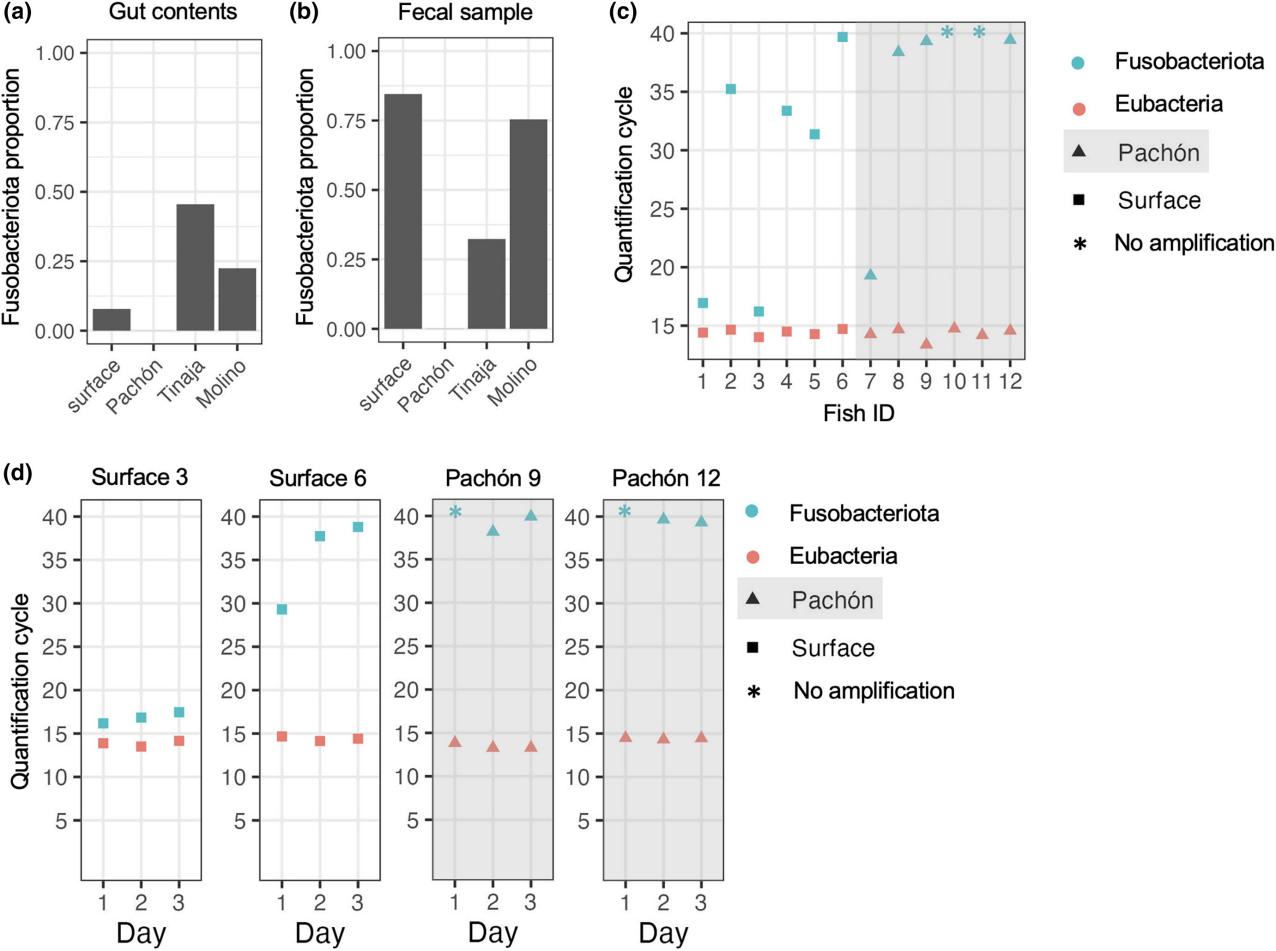
Low abundance of Fusobacteriota in *Astyanax mexicanus* Pachón cavefish can be detected in individual fish over time using fecal samples. (a) Bar graph showing average proportional abundance of Fusobacteriota ASVs in 16s rRNA gene sequencing of intestinal contents of *A. mexicanus* surface fish and cavefish raised in the laboratory (*n* = 35). (b) Bar graph showing proportional abundance of Fusobacteriota ASVs in 16s rRNA gene sequencing in pooled fecal samples from *A. mexicanus* surface fish and cavefish morphotypes raised in the laboratory (*n* = 4). (c) Quantification of Fusobacteriota 16s rRNA genes and Eubacteria 16s rRNA genes in fecal samples from individual 3‐year‐old siblings of the surface fish and Pachón cavefish used for intestinal microbiome sequencing. Greater quantification cycle indicates lower abundance. Eubacteria is not different between samples indicating the total amount of bacterial DNA has been properly controlled. (d) Quantification of Fusobacteriota 16s rRNA genes and Eubacteria 16s rRNA genes in fecal samples collected on three different days from individual 3‐year‐old siblings of surface fish and Pachón cavefish used for microbiome sequencing.

We found that Fusobacteriota was at very low proportional abundance in 16s rRNA gene sequencing data from gut contents and pooled fecal samples of laboratory‐raised Pachón cavefish compared to other morphotypes (Figure 4a,b). Discovering differences in abundance in fecal samples suggested that (1) feces can be used to quantify differences in Fusobacteriota between individuals, and (2) an individual fish could be sampled over time. We collected feces from individual three‐year‐old surface fish and Pachón cavefish that were siblings of the 6‐month‐old fish used for microbiome sequencing. Using quantitative PCR, we found that Fusobacteriota 16s rRNA genes were always detected in the feces of the surface fish (*n* = 6) and were undetectable (*n* = 2/6) or had a higher threshold for detection in the feces of most Pachón cavefish (*n* = 3/4, Figure 4c). Our data suggest that Fusobacteriota abundance varies between individual fish, but that lower abundance in Pachón compared to surface fish is a trait that persists through life. We also found that Fusobacteriota abundance was stable in individual fish sampled at different times; fish with high or low abundance consistently had high or low abundance (Figure 4d). The results demonstrate the feasibility of using proportional abundance of Fusobacteriota as a trait for genetic mapping in *A. mexicanus* to identify host‐driven molecular pathways that shape microbiome composition.

## 4 DISCUSSION

We characterized the intestinal microbiome of *A. mexicanus* surface fish and cavefish morphotypes in their natural habitat and reared under identical conditions in the laboratory to understand (1) if wild cavefish and surface fish have different microbiomes, (2) if the wild microbiomes are represented in the laboratory‐reared fish, and (3) if host evolutionary history alone drives differences in microbiome composition. Our results show that in their natural habitat, surface fish and cavefish are dominated by only a few phyla and there is considerable interindividual variation comparing river‐dwelling and cave‐dwelling fish. We found that the laboratory environment supports the growth of a richer microbial community that contains more of the wild surface fish taxa compared to the wild cavefish taxa. By comparing the microbiomes of surface fish and multiple cavefish morphotypes that were reared in the laboratory, we discovered that host genetics drives differences in microbiome richness and composition and that the microbial community relationships recapitulate the phylogeny of the host. Our study shows that phylosymbiosis can occur within the same species consisting of populations that have adapted to dramatically different habitats. Furthermore, we found consistent and readily quantifiable differences in taxa between *A. mexicanus* morphotypes that can be used to investigate the genetic basis of microbial community structure and how it impacts the development, physiology, metabolism, and behavior of the host.

### 4.1 Drivers of microbiome composition in *A. mexicanus* compared to other fishes

Our study adds to the investigations of what drives microbiome diversity in fishes more broadly. We found that in *A. mexicanus*, evolutionary history alone can account for differences in gut microbiome composition. Most studies on other fish species have found that habitat is the main driver of microbial diversity. For example, a meta‐analysis of 25 fish species revealed that trophic level and salinity were the best predictor of microbiome composition (Sullam et al., 2012). Similarly, a study examining the microbiome of 227 individuals from 85 fish species found that host habitat most strongly shaped the microbiome (Kim et al., 2021). The dominant impact of habitat has also been observed when comparing closely related species; the microbiomes of Cichlids from two lakes cluster by diet (Baldo et al., 2017; Härer et al., 2020). Nevertheless, a role for host phylogeny has been demonstrated in some comparisons. A recent examination of 24 freshwater species from the Yellow River found that diet, location, and host phylogeny predict microbiome composition (Pan et al., 2023).

The studies described above‐compared field caught fish and statistically analyzed the impact of habitat on microbiome composition. In contrast, we controlled the variable of habitat by comparing fish that were spawned in the laboratory, consumed the same diet, and were raised in the same water. A study of a similar design using threespine stickleback (*Gasterosteus aculeatus*) compared the microbiomes of freshwater benthic and freshwater limnetic ecotypes from three different lakes (Rennison et al., 2019). In contrast to what we observed in *A. mexicanus*, the ecotypes did not develop different microbiomes when raised in the same conditions. The harsh cave environment may select for traits that have an outsized effect on microbiome composition compared to the variable environments experienced during the adaptive radiation of other fish species. An emerging theme in predicting microbiome composition is that the relative impact of habitat and evolutionary history depends on the ecological forces that shaped the clade being examined.

### 4.2 Limitations in sampling and methodology

Some studies have shown that age and sex can impact fish microbiome composition (Navarro‐Barrón et al., 2019; Piazzon et al., 2019). Although not significantly different, the field‐collected surface fish were larger on average compared to Pachón cavefish and were all sexually differentiated suggesting they may be older (Figure S1). Due to the limited availability of samples, we were not able to test how these variables may impact microbiome composition. However, in the laboratory, we collected fish that were the same age (6 months old). The lab‐raised morphotypes were not significantly different in weight at the time of collection, although Pachón and Tinaja cavefish weighed more than surface fish and Molino cavefish on average (Figure S1). Interestingly, most Tinaja and Pachón cavefish had undefined sex compared to surface fish and Molino cavefish (Figure S1). Additional sampling of fish across different life stages would help delineate the impact of age, size, and sex on microbiome composition in *A. mexicanus*.

To investigate how host identity impacts microbiome composition, we compared the offspring of surface, Tinaja, Pachón, and Molino *A. mexicanus* that have been maintained in the same laboratory environment on the same diet for multiple generations. We removed spawned embryos from parental tanks, treated them with bleach before hatching, and raised them on the same diet. The morphotypes developed different microbiomes despite experiencing the same environment suggesting host identity is a major driver of microbiome composition. However, we cannot exclude the possibility that bleaching the embryos did not eliminate the influence of the parental microbiome. Vertically transmitted microbes, or microbes that remained associated with the embryos after bleaching could influence the early colonization and subsequent development of the gut microbiota. As stated previously, however, the parents of the fish used in this study were also raised in the laboratory on the same diet. Therefore, if the parental microbiomes differed, it would also be largely driven by the identity of the host. Future studies may utilize germ‐free derivation techniques and inoculation with known microbes to understand the strength of the host‐driven mechanisms that shape microbiome composition in *A. mexicanus*.

### 4.3 Implications for investigating the adaptive benefit of cavefish traits

Our study provides important context for research on the evolution of *A. mexicanus* behavior and metabolism as these traits are known to be influenced by microbiome composition. For example, cavefish have reduced aggression (Rodriguez‐Morales et al., 2022), which can be recapitulated in flies, mice, and hamsters through microbiome manipulation (Gulledge et al., 2023). In addition, cavefish are fat (Xiong et al., 2022) and insulin resistant (Riddle, Aspiras, et al., 2018) which is associated with microbiome changes in fish and mammals (Qin et al., 2012; Turnbaugh et al., 2006). When considering the adaptive benefit of *A. mexicanus* traits recorded in the laboratory, it is important to note that the laboratory environment does not recapitulate either wild habitat (river or cave) and the fish display considerable plasticity in some traits (Bilandžija et al., 2020). We showed that laboratory‐raised fish harbor a more diverse microbiome compared to their field‐caught counterparts and that the laboratory microbiome contains more of the taxa in wild surface fish compared to the wild cavefish. Our results serve as a foundation for understanding how different microbial taxa impact *A. mexicanus* phenotypes which will lead to a better understanding of cavefish evolution.

### 4.4 Functional significance of differences in taxonomic composition between morphotypes

The taxonomic differences we observed between the microbiomes of laboratory‐raised *A. mexicanus* morphotypes have been associated with host traits in other species. For example, we observed a higher ratio Firmicutes to Bacteroidetes ratio (F/B ratio) in cavefish compared to surface fish (Table S4) which has been linked with obesity in mammals (Stojanov et al., 2020). Cavefish are fatter than surface fish (Xiong et al., 2018) and the role for the microbiome in underlying increased adiposity has yet to be explored. We found that Pachón cavefish samples had a high abundance of *Cupriavidus*, which is a genus associated with resistance to copper toxicity (Pan et al., 2021). Whether Pachón cavefish are resistant to copper has not been tested but copper concentrations in the Pachón cave were lower than in the surface river at the time of collection (Table S1). *Cupriavidus* has been found at higher abundance in human patients with type‐2 diabetes compared to healthy individuals (Nah et al., 2019). Interestingly, Pachón cavefish have traits that model diabetes like insulin resistance and higher blood sugar (Riddle, Aspiras, et al., 2018), and a possible connection with the microbiome has not yet been tested.

Another taxonomic difference that shows interesting alignment with Pachón cavefish traits is the near absence of Fusobacteriota of the genus *Cetobacterium* compared to the other morphotypes. *Cetobacterium* is common in the microbiome of herbivorous fish and is thought to assist in carbohydrate metabolism (Tsuchiya et al., 2007). One predominant species, *Cetobacterium somerae*, has been shown to produce vitamin B12 which provides resistance to pathogen infection (Qi et al., 2023). *C. somerae* has also been shown to improve glucose homeostasis when administered to zebrafish, *Danio rerio*, through the production of acetate and modulation of the gut‐brain axis (Wang et al., 2021). Low *Cetobacterium* abundance in Pachón cavefish could therefore be linked with their observed increased sensitivity to infection (Peuß et al., 2020) and impaired glucose homeostasis (Riddle, Aspiras, et al., 2018). In humans, an overabundance of microbes from the same family (*Fusobacteriaceae*) play a role in the progression of diseases like periodontitis, appendicitis, and gastrointestinal cancer (Griffen et al., 2012; Kostic et al., 2012; Swidsinski et al., 2011). For example, an abundance of *Cetobacterium* in combination with other microbes can be used as a diagnostic biomarker for colorectal cancer (Yao et al., 2021). Discovering the genetic basis of low Fusobacteriota abundance in Pachón cavefish may have biomedical relevance in addition to furthering our understanding of cavefish evolution and host–microbe interactions.

### 4.5 *A. mexicanus* as a model for investigating host‐microbiome interactions

Our study stands apart from other investigations on the factors that influence the fish microbiome due to the nature and future promise of the model system. *A. mexicanus* consists of multiple cavefish populations of polyphyletic origin allowing investigations of parallel evolution. We found that host‐driven microbiome differences exist between and within branches of the *A. mexicanus* phylogeny providing multiple natural replicates to investigate the evolution of microbiome composition. In most other species it is not possible to compare divergent populations and a representative ancestor in a controlled laboratory setting. *A. mexicanus* is easy to grow and manipulate in the lab, standardized husbandry protocols are published (Riddle, Martineau, et al., 2018), the genome is sequenced and annotated (Warren et al., 2021), and there are a growing number of resources available to examine and manipulate gene function (Stahl, Jaggard, et al., 2019). Furthermore, methods for deriving germ‐free zebrafish, and inoculating them with specific microbes could be applied to *A. mexicanus* (Melancon et al., 2017). Since post‐larval fish are transparent like zebrafish, the biogeography of the *A. mexicanus* microbiome could be visualized in live fish using fluorescent microbes. Perhaps most importantly, the surface fish and cavefish are interfertile allowing hybridization of surface/cave and cave/cave individuals to investigate the heritability and genetic basis of traits using QTL mapping. It is possible to generate hundreds of F2 hybrids and use them to evaluate complex interactions between host genotype, phenotype, and microbiome community structure. We showed that proportional abundance of Fusobacteriota is very different between surface fish and Pachón cavefish and stable within individuals suggesting it would be a promising taxon to focus on for future QTL studies. In addition to providing insight into the relative impacts of environment and host in determining microbiome composition, our findings establish *A. mexicanus* as an evolutionary model to investigate the molecular mechanisms that mediate host–microbe interactions.

## 5 CONCLUSION

*Astyanax mexicanus* is a powerful model system to investigate the genetic basis of morphological, physiological, and behavioral evolution due to the ability to compare distinct cavefish morphotypes to their extant surface fish ancestor. We were able to discover and define the effects of environment and host evolutionary history on the gut microbiome of this species due to the ability to study the morphotypes in their natural habitat and compare them under the same controlled conditions in the laboratory. We found that the microbiomes of cavefish and surface fish in the wild are dominated by only a few phyla. Fish in the laboratory have a more diverse microbiome that is distinct in composition compared to their wild counterparts. In addition, there are more shared microbes between lab versus wild surface fish compared to lab versus wild cavefish. Importantly, we determined that host genetics alone can drive differences in microbiome diversity and composition by comparing the microbiomes of surface fish and multiple cavefish morphotypes that were reared in the laboratory under identical conditions. Moreover, our data revealed that the differences in microbiome composition mirror the *A. mexicanus* phylogeny showing that host evolutionary history within a single species can shape gut microbial community structure.

## AUTHOR CONTRIBUTIONS

**Misty R. Riddle:** Conceptualization (lead); data curation (lead); formal analysis (lead); investigation (lead); visualization (lead); writing – original draft (lead). **Nguyen K. Nguyen:** Data curation (lead); formal analysis (lead); investigation (supporting); methodology (lead); visualization (lead); writing – review and editing (equal). **Maeve Nave:** Formal analysis (supporting); investigation (supporting); methodology (supporting); writing – review and editing (equal). **Robert Peuß:** Investigation (supporting); methodology (supporting); writing – review and editing (equal). **Ernesto Maldonado:** Investigation (supporting); methodology (supporting); writing – review and editing (equal). **Nicolas Rohner:** Funding acquisition (supporting); resources (supporting); supervision (supporting); writing – review and editing (equal). **Clifford J. Tabin:** Conceptualization (supporting); funding acquisition (lead); resources (lead); writing – review and editing (equal).

## CONFLICT OF INTEREST STATEMENT

The authors declare no competing interests.

## Supporting information


Data S1:

## Data Availability

Sequencing data are available through the Sequence Read Archive (Bio Project ID: PRJNA994970, https://www.ncbi.nlm.nih.gov/bioproject/PRJNA994970). Instructions for downloading the data can be found at https://www.ncbi.nlm.nih.gov/sra/docs/sradownload/. Any additional information required to reanalyze the data reported in this paper is available from the lead contact upon request.

## References

[ece311192-bib-0001] Anderson, J. F. , Johnson, R. C. , Magnarelli, L. A. , Hyde, F. W. , & Andreadis, T. G. (1987). New infectious spirochete isolated from short‐tailed shrews and white‐footed mice. Journal of Clinical Microbiology, 25(8), 1490–1494. 10.1128/jcm.25.8.1490-1494.1987 3305565 PMC269255

[ece311192-bib-0002] Aspiras, A. C. , Rohner, N. , Martineau, B. , Borowsky, R. L. , & Tabin, C. J. (2015). Melanocortin 4 receptor mutations contribute to the adaptation of cavefish to nutrient‐poor conditions. Proceedings of the National Academy of Sciences of the United States of America, 112(31), 9668–9673. 10.1073/pnas.1510802112 26170297 PMC4534248

[ece311192-bib-0003] Baldo, L. , Pretus, J. L. , Riera, J. L. , Musilova, Z. , Bitja Nyom, A. R. , & Salzburger, W. (2017). Convergence of gut microbiotas in the adaptive radiations of African cichlid fishes. ISME Journal, 11(9), 1975–1987. 10.1038/ismej.2017.62 28509910 PMC5560477

[ece311192-bib-0004] Bates, J. M. , Mittge, E. , Kuhlman, J. , Baden, K. N. , Cheesman, S. E. , & Guillemin, K. (2006). Distinct signals from the microbiota promote different aspects of zebrafish gut differentiation. Developmental Biology, 297(2), 374–386. 10.1016/j.ydbio.2006.05.006 16781702

[ece311192-bib-0005] Bilandžija, H. , Hollifield, B. , Steck, M. , Meng, G. , Ng, M. , Koch, A. D. , Gračan, R. , Ćetković, H. , Porter, M. L. , Renner, K. J. , & Jeffery, W. (2020). Phenotypic plasticity as a mechanism of cave colonization and adaptation. eLife, 9, e51830. 10.7554/eLife.51830 32314737 PMC7173965

[ece311192-bib-0006] Bokulich, N. A. , Kaehler, B. D. , Rideout, J. R. , Dillon, M. , Bolyen, E. , Knight, R. , Huttley, G. A. , & Gregory Caporaso, J. (2018). Optimizing taxonomic classification of marker‐gene amplicon sequences with QIIME 2's q2‐feature‐classifier plugin. Microbiome, 6(1), 90. 10.1186/s40168-018-0470-z 29773078 PMC5956843

[ece311192-bib-0007] Bolyen, E. , Rideout, J. R. , Dillon, M. R. , Bokulich, N. A. , Abnet, C. C. , Al‐Ghalith, G. A. , Alexander, H. , Alm, E. J. , Arumugam, M. , Asnicar, F. , Bai, Y. , Bisanz, J. E. , Bittinger, K. , Brejnrod, A. , Brislawn, C. J. , Brown, C. T. , Callahan, B. J. , Caraballo‐Rodríguez, A. M. , Chase, J. , … Caporaso, J. G. (2019). Reproducible, interactive, scalable and extensible microbiome data science using QIIME 2. Nature Biotechnology, 37(8), 852–857. 10.1038/s41587-019-0209-9 PMC7015180 31341288

[ece311192-bib-0008] Boutaga, K. , Winkelhoff, A. J. , Vandenbroucke‐Grauls, C. M. J. E. , & Savelkoul, P. H. M. (2005). Periodontal pathogens: A quantitative comparison of anaerobic culture and real‐time PCR. FEMS Immunology and Medical Microbiology, 45(2), 191–199. 10.1016/j.femsim.2005.03.011 15919188

[ece311192-bib-0009] Brooks, A. W. , Kohl, K. D. , Brucker, R. M. , van Opstal, E. J. , & Bordenstein, S. R. (2016). Phylosymbiosis: Relationships and functional effects of microbial communities across host evolutionary history. PLoS Biology, 14(11), e2000225. 10.1371/journal.pbio.2000225 27861590 PMC5115861

[ece311192-bib-0010] Brown, R. M. , Wiens, G. D. , & Salinas, I. (2019). Analysis of the gut and gill microbiome of resistant and susceptible lines of rainbow trout (*Oncorhynchus mykiss*). Fish & Shellfish Immunology, 86, 497–506. 10.1016/j.fsi.2018.11.079 30513381 PMC8040288

[ece311192-bib-0011] Callahan, B. J. , McMurdie, P. J. , Rosen, M. J. , Han, A. W. , Johnson, A. J. A. , & Holmes, S. P. (2016). DADA2: High‐resolution sample inference from Illumina amplicon data. Nature Methods, 13(7), 581–583. 10.1038/nmeth.3869 27214047 PMC4927377

[ece311192-bib-0012] Carmody, R. N. , Gerber, G. K. , Luevano, J. M. , Gatti, D. M. , Somes, L. , Svenson, K. L. , & Turnbaugh, P. J. (2015). Diet dominates host genotype in shaping the murine gut microbiota. Cell Host & Microbe, 17(1), 72–84. 10.1016/j.chom.2014.11.010 25532804 PMC4297240

[ece311192-bib-0013] Corless, C. E. , Guiver, M. , Borrow, R. , Edwards‐Jones, V. , Kaczmarski, E. B. , & Fox, A. J. (2000). Contamination and sensitivity issues with a real‐time universal 16S rRNA PCR. Journal of Clinical Microbiology, 38(5), 1747–1752. 10.1128/JCM.38.5.1747-1752.2000 10790092 PMC86577

[ece311192-bib-0014] Dahm, R. , & Nüsslein‐Volhard, C. (2002). Zebrafish: A practical approach. Oxford University Press.

[ece311192-bib-0015] David, L. A. , Maurice, C. F. , Carmody, R. N. , Gootenberg, D. B. , Button, J. E. , Wolfe, B. E. , Ling, A. V. , Devlin, A. S. , Varma, Y. , Fischbach, M. A. , Biddinger, S. B. , Dutton, R. J. , & Turnbaugh, P. J. (2014). Diet rapidly and reproducibly alters the human gut microbiome. Nature, 505(7484), 559–563. 10.1038/nature12820 24336217 PMC3957428

[ece311192-bib-0016] Duboué, E. R. , Keene, A. C. , & Borowsky, R. L. (2011). Evolutionary convergence on sleep loss in cavefish populations. Current Biology, 21(8), 671–676. 10.1016/j.cub.2011.03.020 21474315

[ece311192-bib-0017] Elliott, W. R. (2016). Cave biodiversity and ecology of the Sierra de El Abra region. In Biology and evolution of the Mexican cavefish (pp. 59–76). Elsevier. 10.1016/B978-0-12-802148-4.00003-7

[ece311192-bib-0018] Espinasa, L. , Bonaroti, N. , Wong, J. , Pottin, K. , Queinnec, E. , & Rétaux, S. (2017). Contrasting feeding habits of post‐larval and adult Astyanax cavefish. Subterranean Biology, 21(1), 1–17. 10.3897/subtbiol.21.11046

[ece311192-bib-0019] Ganal‐Vonarburg, S. C. , Hornef, M. W. , & Macpherson, A. J. (2020). Microbial‐host molecular exchange and its functional consequences in early mammalian life. Science, 368(6491), 604–607. 10.1126/science.aba0478 32381716

[ece311192-bib-0020] Gould, A. L. , Zhang, V. , Lamberti, L. , Jones, E. W. , Obadia, B. , Korasidis, N. , Gavryushkin, A. , Carlson, J. M. , Beerenwinkel, N. , & Ludington, W. B. (2018). Microbiome interactions shape host fitness. Proceedings of the National Academy of Sciences of the United States of America, 115(51), E11951–E11960. 10.1073/pnas.1809349115 30510004 PMC6304949

[ece311192-bib-0021] Greene, L. K. , Williams, C. V. , Junge, R. E. , Mahefarisoa, K. L. , Rajaonarivelo, T. , Rakotondrainibe, H. , O'Connell, T. M. , & Drea, C. M. (2020). A role for gut microbiota in host niche differentiation. The ISME Journal, 14(7), 1675–1687. 10.1038/s41396-020-0640-4 32238913 PMC7305313

[ece311192-bib-0022] Griffen, A. L. , Beall, C. J. , Campbell, J. H. , Firestone, N. D. , Kumar, P. S. , Yang, Z. K. , Podar, M. , & Leys, E. J. (2012). Distinct and complex bacterial profiles in human periodontitis and health revealed by 16S pyrosequencing. The ISME Journal, 6(6), 1176–1185. 10.1038/ismej.2011.191 22170420 PMC3358035

[ece311192-bib-0023] Gross, J. B. , Krutzler, A. J. , & Carlson, B. M. (2014). Complex craniofacial changes in blind cave‐dwelling fish are mediated by genetically symmetric and asymmetric loci. Genetics, 196(4), 1303–1319. 10.1534/genetics.114.161661 24496009 PMC3982692

[ece311192-bib-0024] Gulledge, L. , Oyebode, D. , & Donaldson, J. R. (2023). The influence of the microbiome on aggressive behavior: An insight into age‐related aggression. FEMS Microbiology Letters, 370, fnac114. 10.1093/femsle/fnac114 36881728

[ece311192-bib-0025] Härer, A. , Torres‐Dowdall, J. , Rometsch, S. J. , Yohannes, E. , Machado‐Schiaffino, G. , & Meyer, A. (2020). Parallel and non‐parallel changes of the gut microbiota during trophic diversification in repeated young adaptive radiations of sympatric cichlid fish. Microbiome, 8(1), 149. 10.1186/s40168-020-00897-8 33121541 PMC7597055

[ece311192-bib-0026] Herman, A. , Brandvain, Y. , Weagley, J. , Jeffery, W. R. , Keene, A. C. , Kono, T. J. Y. , Bilandžija, H. , Borowsky, R. , Espinasa, L. , O'Quin, K. , Ornelas‐García, C. P. , Yoshizawa, M. , Carlson, B. , Maldonado, E. , Gross, J. B. , Cartwright, R. A. , Rohner, N. , Warren, W. C. , & McGaugh, S. E. (2018). The role of gene flow in rapid and repeated evolution of cave‐related traits in Mexican tetra, *Astyanax mexicanus* . Molecular Ecology, 27(22), 4397–4416. 10.1111/mec.14877 30252986 PMC6261294

[ece311192-bib-0027] Jia, Y. , Jin, S. , Hu, K. , Geng, L. , Han, C. , Kang, R. , Pang, Y. , Ling, E. , Tan, E. K. , Pan, Y. , & Liu, W. (2021). Gut microbiome modulates drosophila aggression through octopamine signaling. Nature Communications, 12(1), 2698. 10.1038/s41467-021-23041-y PMC8113466 33976215

[ece311192-bib-0028] Kemis, J. H. , Linke, V. , Barrett, K. L. , Boehm, F. J. , Traeger, L. L. , Keller, M. P. , Rabaglia, M. E. , Schueler, K. L. , Stapleton, D. S. , Gatti, D. M. , Churchill, G. A. , Amador‐Noguez, D. , Russell, J. D. , Yandell, B. S. , Broman, K. W. , Coon, J. J. , Attie, A. D. , & Rey, F. E. (2019). Genetic determinants of gut microbiota composition and bile acid profiles in mice. PLoS Genetics, 15(8), e1008073. 10.1371/journal.pgen.1008073 31465442 PMC6715156

[ece311192-bib-0029] Kim, P. S. , Shin, N.‐R. , Lee, J.‐B. , Kim, M.‐S. , Whon, T. W. , Hyun, D.‐W. , Yun, J.‐H. , Jung, M.‐J. , Kim, J. Y. , & Bae, J.‐W. (2021). Host habitat is the major determinant of the gut microbiome of fish. Microbiome, 9(1), 166. 10.1186/s40168-021-01113-x 34332628 PMC8325807

[ece311192-bib-0030] Klaassen, H. , Wang, Y. , Adamski, K. , Rohner, N. , & Kowalko, J. E. (2018). CRISPR mutagenesis confirms the role of oca2 in melanin pigmentation in *Astyanax mexicanus* . Developmental Biology, 441, 313–318. 10.1016/j.ydbio.2018.03.014 29555241

[ece311192-bib-0031] Kostic, A. D. , Chun, E. , Robertson, L. , Glickman, J. N. , Gallini, C. A. , Michaud, M. , Clancy, T. E. , Chung, D. C. , Lochhead, P. , Hold, G. L. , El‐Omar, E. M. , Brenner, D. , Fuchs, C. S. , Meyerson, M. , & Garrett, W. S. (2013). *Fusobacterium nucleatum* potentiates intestinal tumorigenesis and modulates the tumor‐immune microenvironment. Cell Host & Microbe, 14(2), 207–215. 10.1016/j.chom.2013.07.007 23954159 PMC3772512

[ece311192-bib-0032] Kostic, A. D. , Gevers, D. , Pedamallu, C. S. , Michaud, M. , Duke, F. , Earl, A. M. , Ojesina, A. I. , Jung, J. , Bass, A. J. , Tabernero, J. , Baselga, J. , Liu, C. , Shivdasani, R. A. , Ogino, S. , Birren, B. W. , Huttenhower, C. , Garrett, W. S. , & Meyerson, M. (2012). Genomic analysis identifies association of fusobacterium with colorectal carcinoma. Genome Research, 22(2), 292–298. 10.1101/gr.126573.111 22009990 PMC3266036

[ece311192-bib-0033] Kowalko, J. E. , Rohner, N. , Linden, T. A. , Rompani, S. B. , Warren, W. C. , Borowsky, R. , Tabin, C. J. , Jeffery, W. R. , & Yoshizawa, M. (2013). Convergence in feeding posture occurs through different genetic loci in independently evolved cave populations of *Astyanax mexicanus* . Proceedings of the National Academy of Sciences of the United States of America, 110(42), 16933–16938. 10.1073/pnas.1317192110 24085851 PMC3801050

[ece311192-bib-0034] Kowalko, J. E. , Rohner, N. , Rompani, S. B. , Peterson, B. K. , Linden, T. A. , Yoshizawa, M. , Kay, E. H. , Weber, J. , Hoekstra, H. E. , Jeffery, W. R. , Borowsky, R. , & Tabin, C. J. (2013). Loss of schooling behavior in cavefish through sight‐dependent and sight‐independent mechanisms. Current Biology, 23(19), 1874–1883. 10.1016/j.cub.2013.07.056 24035545 PMC3904651

[ece311192-bib-0092] Kozich, J. J. , Westcott, S. L. , Baxter, N. T. , Highlander, S. K. , & Schloss, P. D. (2013). Development of a dual‐index sequencing strategy and curation pipeline for analyzing amplicon sequence data on the MiSeq Illumina sequencing platform. Applied and Environmental Microbiology, 79(17), 5112–5120. 10.1128/AEM.01043-13 23793624 PMC3753973

[ece311192-bib-0035] Krishnan, J. , & Rohner, N. (2017). Cavefish and the basis for eye loss. Philosophical Transactions of the Royal Society, B: Biological Sciences, 372(1713), 20150487. 10.1098/rstb.2015.0487 PMC5182419 27994128

[ece311192-bib-0036] Lahti, L. , & Shetty, S. (2017). Microbiome R package . http://microbiome.github.io

[ece311192-bib-0037] Leigh, S. C. , Catabay, C. , & German, D. P. (2022). Sustained changes in digestive physiology and microbiome across sequential generations of zebrafish fed different diets. Comparative Biochemistry and Physiology Part A: Molecular & Integrative Physiology, 273, 111285. 10.1016/j.cbpa.2022.111285 35961610

[ece311192-bib-0038] Li, T. , Raza, S. H. A. , Yang, B. , Sun, Y. , Wang, G. , Sun, W. , Qian, A. , Wang, C. , Kang, Y. , & Shan, X. (2020). Aeromonas veronii infection in commercial freshwater fish: A potential threat to public health. Animals, 10(4), 608. 10.3390/ani10040608 32252334 PMC7222775

[ece311192-bib-0039] Li, Y. , Bruni, L. , Jaramillo‐Torres, A. , Gajardo, K. , Kortner, T. M. , & Krogdahl, Å. (2021). Differential response of digesta‐ and mucosa‐associated intestinal microbiota to dietary insect meal during the seawater phase of Atlantic salmon. Animal Microbiome, 3(1), 8. 10.1186/s42523-020-00071-3 33500000 PMC7934271

[ece311192-bib-0040] Lim, S. J. , & Bordenstein, S. R. (2020). An introduction to phylosymbiosis. Proceedings of the Royal Society B: Biological Sciences, 287(1922), 20192900. 10.1098/rspb.2019.2900 PMC7126058 32126958

[ece311192-bib-0041] Lozupone, C. , Lladser, M. E. , Knights, D. , Stombaugh, J. , & Knight, R. (2011). UniFrac: An effective distance metric for microbial community comparison. The ISME Journal, 5(2), 169–172. 10.1038/ismej.2010.133 20827291 PMC3105689

[ece311192-bib-0042] McMurdie, P. J. , & Holmes, S. (2013). Phyloseq: An R package for reproducible interactive analysis and graphics of microbiome census data. PLoS ONE, 8(4), e61217. 10.1371/journal.pone.0061217 23630581 PMC3632530

[ece311192-bib-0043] Melancon, E. , De La Torre, G. , Canny, S. , Sichel, S. , Kelly, M. , Wiles, T. J. , Rawls, J. F. , Eisen, J. S. , & Guillemin, K. (2017). Best practices for germ‐free derivation and gnotobiotic zebrafish husbandry. Methods in Cell Biology, 138, 61–100. 10.1016/bs.mcb.2016.11.005 28129860 PMC5568843

[ece311192-bib-0044] Mills, D. V. G. (1989). The tetra encyclopedia of freshwater tropical aquarium fishes (p. 208). Tetra Press.

[ece311192-bib-0045] Nah, G. , Park, S. C. , Kim, K. , Kim, S. , Park, J. , Lee, S. , & Won, S. (2019). Type‐2 diabetics reduces spatial variation of microbiome based on Extracellur vesicles from gut microbes across human body. Scientific Reports, 9(1), 20136. 10.1038/s41598-019-56662-x 31882892 PMC6934622

[ece311192-bib-0046] Navarro‐Barrón, E. , Hernández, C. , Llera‐Herrera, R. , García‐Gasca, A. , & Gomez‐Gil, B. (2019). Overfeeding a high‐fat diet promotes sex‐specific alterations on the gut microbiota of the zebrafish (*Danio rerio*). Zebrafish, 16, 268–279. 10.1089/zeb.2018.1648 30964393

[ece311192-bib-0047] Oksanen, J. , Blanchet, F. G. , Kindt, R. , Legendre, P. , Minchin, P. , O'Hara, B. , Simpson, G. , Solymos, P. , Stevens, H. , & Wagner, H. (2015). Vegan: Community ecology package . R Package Version 2.2‐1, 2, 1‐2.

[ece311192-bib-0048] Olsen, L. , Levy, M. , Medley, J. K. , Hassan, H. , Miller, B. , Alexander, R. , Wilcock, E. , Yi, K. , Florens, L. , Weaver, K. , McKinney, S. A. , Peuß, R. , Persons, J. , Kenzior, A. , Maldonado, E. , Delventhal, K. , Gluesenkamp, A. , Mager, E. , Coughlin, D. , & Rohner, N. (2023). Metabolic reprogramming underlies cavefish muscular endurance despite loss of muscle mass and contractility. Proceedings of the National Academy of Sciences of the United States of America, 120(5), e2204427120. 10.1073/pnas.2204427120 36693105 PMC9945943

[ece311192-bib-0049] Ornelas‐García, C. P. , & Pedraza‐Lara, C. (2015). Phylogeny and evolutionary history of *Astyanax mexicanus* . In A. C. Keene , M. Yoshizawa , & S. E. McGaugh (Eds.), Biology and evolution of the Mexican cavefish (pp. 77–90). Elsevier Inc. 10.1016/B978-0-12-802148-4.00004-9

[ece311192-bib-0050] Ornelas‐García, P. , Pajares, S. , Sosa‐Jiménez, V. M. , Rétaux, S. , & Miranda‐Gamboa, R. A. (2018). Microbiome differences between river‐dwelling and cave‐adapted populations of the fish *Astyanax mexicanus* (De Filippi, 1853). PeerJ, 6, e5906. 10.7717/peerj.5906 30425894 PMC6228550

[ece311192-bib-0051] Pan, B. , Han, X. , Yu, K. , Sun, H. , Mu, R. , & Lian, C.‐A. (2023). Geographical distance, host evolutionary history and diet drive gut microbiome diversity of fish across the Yellow River. Molecular Ecology, 32(5), 1183–1196. 10.1111/mec.16812 36478318

[ece311192-bib-0052] Pan, Y. , Li, Z. , Zhou, J. , Wang, Q. , Xu, H. , & Mou, Z. (2021). Cupriavidus in the intestinal microbiota of Tibet endemic fish *Glyptosternum maculatum* can help it adapt to habitat of the Qinghai Tibet plateau. BMC Veterinary Research, 17(1), 377. 10.1186/s12917-021-03092-5 34876102 PMC8650323

[ece311192-bib-0053] Peuß, R. , Box, A. C. , Chen, S. , Wang, Y. , Tsuchiya, D. , Persons, J. L. , Kenzior, A. , Maldonado, E. , Krishnan, J. , Scharsack, J. P. , Slaughter, B. D. , & Rohner, N. (2020). Adaptation to low parasite abundance affects immune investment and immunopathological responses of cavefish. Nature Ecology & Evolution, 4(10), 1416–1430. 10.1038/s41559-020-1234-2 32690906 PMC11062081

[ece311192-bib-0054] Peuß, R. , Zakibe, Z. , Krishnan, J. , Merryman, M. S. , Baumann, D. P. , & Rohner, N. (2019). Gamete collection and in vitro fertilization of *Astyanax mexicanus* . Journal of Visualized Experiments: JoVE, 147, 1–6. 10.3791/59334 31180353

[ece311192-bib-0055] Piazzon, M. C. , Naya‐Català, F. , Simó‐Mirabet, P. , Picard‐Sánchez, A. , Roig, F. J. , Calduch‐Giner, J. A. , Sitjà‐Bobadilla, A. , & Pérez‐Sánchez, J. (2019). Sex, age, and bacteria: How the intestinal microbiota is modulated in a protandrous hermaphrodite fish. Frontiers in Microbiology, 10, 2512. 10.3389/fmicb.2019.02512 31736931 PMC6834695

[ece311192-bib-0093] Ponnimbaduge Perera, P. , Perez Guerra, D. , & Riddle, M. R. (2023). The Mexican Tetra, *Astyanax mexicanus*, as a model system in cell and developmental biology. Annual Review of Cell and Developmental Biology, 39(1), 23–44. 10.1146/annurev-cellbio-012023-014003 37437210

[ece311192-bib-0056] Protas, M. , Conrad, M. , Gross, J. B. , Tabin, C. , & Borowsky, R. (2007). Regressive evolution in the Mexican cave tetra, *Astyanax mexicanus* . Current Biology, 17(5), 452–454. 10.1016/j.cub.2007.01.051 17306543 PMC2570642

[ece311192-bib-0057] Protas, M. E. , Hersey, C. , Kochanek, D. , Zhou, Y. , Wilkens, H. , Jeffery, W. R. , Zon, L. I. , Borowsky, R. , & Tabin, C. J. (2006). Genetic analysis of cavefish reveals molecular convergence in the evolution of albinism. Nature Genetics, 38(1), 107–111. 10.1038/ng1700 16341223

[ece311192-bib-0058] Qi, X. , Zhang, Y. , Zhang, Y. , Luo, F. , Song, K. , Wang, G. , & Ling, F. (2023). Vitamin B12 produced by *Cetobacterium somerae* improves host resistance against pathogen infection through strengthening the interactions within gut microbiota. Microbiome, 11(1), 135. 10.1186/s40168-023-01574-2 37322528 PMC10268390

[ece311192-bib-0059] Qin, J. , Li, Y. , Cai, Z. , Li, S. , Zhu, J. , Zhang, F. , Liang, S. , Zhang, W. , Guan, Y. , Shen, D. , Peng, Y. , Zhang, D. , Jie, Z. , Wu, W. , Qin, Y. , Xue, W. , Li, J. , Han, L. , Lu, D. , … Wang, J. (2012). A metagenome‐wide association study of gut microbiota in type 2 diabetes. Nature, 490(7418), 55–60. 10.1038/nature11450 23023125

[ece311192-bib-0060] Quast, C. , Pruesse, E. , Yilmaz, P. , Gerken, J. , Schweer, T. , Yarza, P. , Peplies, J. , & Glöckner, F. O. (2013). The SILVA ribosomal RNA gene database project: Improved data processing and web‐based tools. Nucleic Acids Research, 41(Database issue), D590–D596. 10.1093/nar/gks1219 23193283 PMC3531112

[ece311192-bib-0061] R‐Core‐Team . (2019). R: A language and environment for statistical computing. R Foundation for Statistical Computing. https://www.r‐project.org/

[ece311192-bib-0062] Rennison, D. J. , Rudman, S. M. , & Schluter, D. (2019). Parallel changes in gut microbiome composition and function during colonization, local adaptation and ecological speciation. Proceedings of the Royal Society B: Biological Sciences, 286(1916), 20191911. 10.1098/rspb.2019.1911 PMC6939261 31795865

[ece311192-bib-0063] Riddle, M. , Martineau, B. , Peavey, M. , & Tabin, C. (2018). Raising the Mexican tetra *Astyanax mexicanus* for analysis of post‐larval phenotypes and whole‐mount immunohistochemistry. Journal of Visualized Experiments, 142. 10.3791/58972 PMC6590686 30638199

[ece311192-bib-0064] Riddle, M. R. , Aspiras, A. , Damen, F. , McGaugh, S. , Tabin, J. A. , & Tabin, C. J. (2021). Genetic mapping of metabolic traits in the blind Mexican cavefish reveals sex‐dependent quantitative trait loci associated with cave adaptation. BMC Ecology and Evolution, 21(1), 94. 10.1186/s12862-021-01823-8 34020589 PMC8139031

[ece311192-bib-0065] Riddle, M. R. , Aspiras, A. C. , Gaudenz, K. , Peuß, R. , Sung, J. Y. , Martineau, B. , Peavey, M. , Box, A. C. , Tabin, J. A. , McGaugh, S. , Borowsky, R. , Tabin, C. J. , & Rohner, N. (2018). Insulin resistance in cavefish as an adaptation to a nutrient‐limited environment. Nature, 555(7698), 647–651. 10.1038/nature26136 29562229 PMC5989729

[ece311192-bib-0066] Robeson, M. S. , O'Rourke, D. R. , Kaehler, B. D. , Ziemski, M. , Dillon, M. R. , Foster, J. T. , & Bokulich, N. A. (2021). RESCRIPt: Reproducible sequence taxonomy reference database management. PLoS Computational Biology, 17(11), e1009581. 10.1371/journal.pcbi.1009581 34748542 PMC8601625

[ece311192-bib-0067] Rodriguez‐Morales, R. , Gonzalez‐Lerma, P. , Yuiska, A. , Han, J. H. , Guerra, Y. , Crisostomo, L. , Keene, A. C. , Duboue, E. R. , & Kowalko, J. E. (2022). Convergence on reduced aggression through shared behavioral traits in multiple populations of *Astyanax mexicanus* . BMC Ecology and Evolution, 22(1), 116. 10.1186/s12862-022-02069-8 36241984 PMC9563175

[ece311192-bib-0068] Roeselers, G. , Mittge, E. K. , Stephens, W. Z. , Parichy, D. M. , Cavanaugh, C. M. , Guillemin, K. , & Rawls, J. F. (2011). Evidence for a core gut microbiota in the zebrafish. ISME Journal, 5(10), 1595–1608. 10.1038/ismej.2011.38 21472014 PMC3176511

[ece311192-bib-0069] Rolig, A. S. , Sweeney, E. G. , Kaye, L. E. , DeSantis, M. D. , Perkins, A. , Banse, A. V. , Hamilton, M. K. , & Guillemin, K. (2018). A bacterial immunomodulatory protein with lipocalin‐like domains facilitates host–bacteria mutualism in larval zebrafish. eLife, 7, e37172. 10.7554/eLife.37172 30398151 PMC6219842

[ece311192-bib-0070] Santacruz, A. , Hernández‐Mena, D. , Miranda‐Gamboa, R. , Pérez‐Ponce De León, G. , & Patricia Ornelas‐García, C. (2023). Host‐parasite interactions in perpetual darkness: Macroparasite diversity in the cavefish *Astyanax mexicanus* . Zoological Research, 44(4), 782–792. 10.24272/j.issn.2095-8137.2022.376 37464936 PMC10415763

[ece311192-bib-0071] Schliep, K. P. (2011). Phangorn: Phylogenetic analysis in R. Bioinformatics, 27(4), 592–593. 10.1093/bioinformatics/btq706 21169378 PMC3035803

[ece311192-bib-0072] Stahl, B. A. , Jaggard, J. B. , Chin, J. S. R. , Kowalko, J. E. , Keene, A. C. , & Duboué, E. R. (2019). Manipulation of gene function in Mexican cavefish. Journal of Visualized Experiments, 146. 10.3791/59093 31058898

[ece311192-bib-0073] Stahl, B. A. , Peuß, R. , McDole, B. , Kenzior, A. , Jaggard, J. B. , Gaudenz, K. , Krishnan, J. , McGaugh, S. E. , Duboue, E. R. , Keene, A. C. , & Rohner, N. (2019). Stable transgenesis in *Astyanax mexicanus* using the Tol2 transposase system. Developmental Dynamics, 248(8), 679–687. 10.1002/dvdy.32 30938001 PMC6675643

[ece311192-bib-0074] Staubach, F. , Baines, J. F. , Künzel, S. , Bik, E. M. , & Petrov, D. A. (2013). Host species and environmental effects on bacterial communities associated with drosophila in the laboratory and in the natural environment. PLoS ONE, 8(8), e70749. 10.1371/journal.pone.0070749 23967097 PMC3742674

[ece311192-bib-0075] Stephens, W. Z. , Burns, A. R. , Stagaman, K. , Wong, S. , Rawls, J. F. , Guillemin, K. , & Bohannan, B. J. M. (2016). The composition of the zebrafish intestinal microbial community varies across development. The ISME Journal, 10(3), 644–654. 10.1038/ismej.2015.140 26339860 PMC4817687

[ece311192-bib-0076] Stojanov, S. , Berlec, A. , & Štrukelj, B. (2020). The influence of probiotics on the firmicutes/Bacteroidetes ratio in the treatment of obesity and inflammatory bowel disease. Microorganisms, 8(11), 1715. 10.3390/microorganisms8111715 33139627 PMC7692443

[ece311192-bib-0077] Sullam, K. E. , Essinger, S. D. , Lozupone, C. A. , O'connor, M. P. , Rosen, G. L. , Knight, R. , Kilham, S. S. , & Russell, J. A. (2012). Environmental and ecological factors that shape the gut bacterial communities of fish: A meta‐analysis. Molecular Ecology, 21(13), 3363–3378. 10.1111/j.1365-294X.2012.05552.x 22486918 PMC3882143

[ece311192-bib-0078] Swidsinski, A. , Dorffel, Y. , Loening‐Baucke, V. , Theissig, F. , Ruckert, J. C. , Ismail, M. , Rau, W. A. , Gaschler, D. , Weizenegger, M. , Kuhn, S. , Schilling, J. , & Dorffel, W. V. (2011). Acute appendicitis is characterised by local invasion with *Fusobacterium nucleatum*/*necrophorum* . Gut, 60(1), 34–40. 10.1136/gut.2009.191320 19926616

[ece311192-bib-0079] Tabin, J. A. , Aspiras, A. , Martineau, B. , Riddle, M. , Kowalko, J. , Borowsky, R. , Rohner, N. , & Tabin, C. J. (2018). Temperature preference of cave and surface populations of *Astyanax mexicanus* . Developmental Biology, 441(2), 338–344. 10.1016/j.ydbio.2018.04.017 29704470 PMC6119108

[ece311192-bib-0080] Tapia‐Paniagua, S. T. , Vidal, S. , Lobo, C. , Prieto‐Álamo, M. J. , Jurado, J. , Cordero, H. , Cerezuela, R. , García de la Banda, I. , Esteban, M. A. , Balebona, M. C. , & Moriñigo, M. A. (2014). The treatment with the probiotic *Shewanella putrefaciens* Pdp11 of specimens of *Solea senegalensis* exposed to high stocking densities to enhance their resistance to disease. Fish & Shellfish Immunology, 41(2), 209–221. 10.1016/j.fsi.2014.08.019 25149590

[ece311192-bib-0081] Tsuchiya, C. , Sakata, T. , & Sugita, H. (2007). Novel ecological niche of *Cetobacterium somerae*, an anaerobic bacterium in the intestinal tracts of freshwater fish. Letters in Applied Microbiology, 46(1), 43–48. 10.1111/j.1472-765X.2007.02258.x 17944860

[ece311192-bib-0082] Turnbaugh, P. J. , Ley, R. E. , Mahowald, M. A. , Magrini, V. , Mardis, E. R. , & Gordon, J. I. (2006). An obesity‐associated gut microbiome with increased capacity for energy harvest. Nature, 444(7122), 1027–1031. 10.1038/nature05414 17183312

[ece311192-bib-0083] Wang, A. , Zhang, Z. , Ding, Q. , Yang, Y. , Bindelle, J. , Ran, C. , & Zhou, Z. (2021). Intestinal *Cetobacterium* and acetate modify glucose homeostasis via parasympathetic activation in zebrafish. Gut Microbes, 13(1), 1–15. 10.1080/19490976.2021.1900996 PMC8043178 33840371

[ece311192-bib-0084] Warren, W. C. , Boggs, T. E. , Borowsky, R. , Carlson, B. M. , Ferrufino, E. , Gross, J. B. , Hillier, L. , Hu, Z. , Keene, A. C. , Kenzior, A. , Kowalko, J. E. , Tomlinson, C. , Kremitzki, M. , Lemieux, M. E. , Graves‐Lindsay, T. , McGaugh, S. E. , Miller, J. T. , Mommersteeg, M. T. M. , Moran, R. L. , … Rohner, N. (2021). A chromosome‐level genome of *Astyanax mexicanus* surface fish for comparing population‐specific genetic differences contributing to trait evolution. Nature Communications, 12(1), 1447. 10.1038/s41467-021-21733-z PMC7933363 33664263

[ece311192-bib-0085] Wickham, H. (2016). ggplot2: Elegant graphics for data analysis. Springer‐Verlag New York.

[ece311192-bib-0086] Wilson, E. J. , Tobler, M. , Riesch, R. , Martínez‐García, L. , & García‐De León, F. J. (2021). Natural history and trophic ecology of three populations of the Mexican cavefish, *Astyanax mexicanus* . Environmental Biology of Fishes, 104(11), 1461–1474. 10.1007/s10641-021-01163-y

[ece311192-bib-0087] Xiong, S. , Krishnan, J. , Peuß, R. , & Rohner, N. (2018). Early adipogenesis contributes to excess fat accumulation in cave populations of *Astyanax mexicanus* . Developmental Biology, 441(2), 297–304. 10.1016/j.ydbio.2018.06.003 29883659

[ece311192-bib-0088] Xiong, S. , Wang, W. , Kenzior, A. , Olsen, L. , Krishnan, J. , Persons, J. , Medley, K. , Peuß, R. , Wang, Y. , Chen, S. , Zhang, N. , Thomas, N. , Miles, J. M. , Alvarado, A. S. , & Rohner, N. (2022). Enhanced lipogenesis through Pparγ helps cavefish adapt to food scarcity. Current Biology, 32(10), 2272–2280.e6. 10.1016/j.cub.2022.03.038 35390280 PMC9133166

[ece311192-bib-0089] Yao, Y. , Ni, H. , Wang, X. , Xu, Q. , Zhang, J. , Jiang, L. , Wang, B. , Song, S. , & Zhu, X. (2021). A new biomarker of fecal bacteria for non‐invasive diagnosis of colorectal cancer. Frontiers in Cellular and Infection Microbiology, 11, 744049. 10.3389/fcimb.2021.744049 34976850 PMC8719628

[ece311192-bib-0090] Yu, G. (2020). Using ggtree to visualize data on tree‐like structures. Current Protocols in Bioinformatics, 69(1), e96. 10.1002/cpbi.96 32162851

[ece311192-bib-0091] Zhang, Q. , Linke, V. , Overmyer, K. A. , Traeger, L. L. , Kasahara, K. , Miller, I. J. , Manson, D. E. , Polaske, T. J. , Kerby, R. L. , Kemis, J. H. , Trujillo, E. A. , Reddy, T. R. , Russell, J. D. , Schueler, K. L. , Stapleton, D. S. , Rabaglia, M. E. , Seldin, M. , Gatti, D. M. , Keele, G. R. , … Rey, F. E. (2023). Genetic mapping of microbial and host traits reveals production of immunomodulatory lipids by *Akkermansia muciniphila* in the murine gut. Nature Microbiology, 8(3), 424–440. 10.1038/s41564-023-01326-w PMC9981464 36759753

