# Correction to “Host evolution shapes gut microbiome composition in *Astyanax mexicanus*”

**DOI:** 10.1002/ece3.11545

**Published:** 2024-06-25

Riddle MR, Nguyen NK, Nave M, Peuss R, Maldonado E, Rohner N, et al. Host evolution shapes gut microbiome composition in *Astyanax mexicanus*. Ecology and evolution. 2024;14(4):e11192.

The funding source of one of the co‐ authors, Robert Peuß, is missing in the above‐ mentioned publication. This correction adds the following sentence to the end of the acknowledgment section:

RP was partly funded by the German Research Foundation (DFG) as part of the CRC TRR 212 (NC^3^)—project number: 316099922; individual project number: 471673683.

