# Supplementary material for: Host evolution shapes gut microbiome composition in Astyanax mexicanus
Source: Ecol Evol. 2024 Apr 1;14(4):e11192. doi: 10.1002/ece3.11192 (PMC10985381; doi:10.1002/ece3.11192)
Supplement: Supplementary file 1 — Data S1: [file ECE3-14-e11192-s001.docx]

**Supporting Information**

**
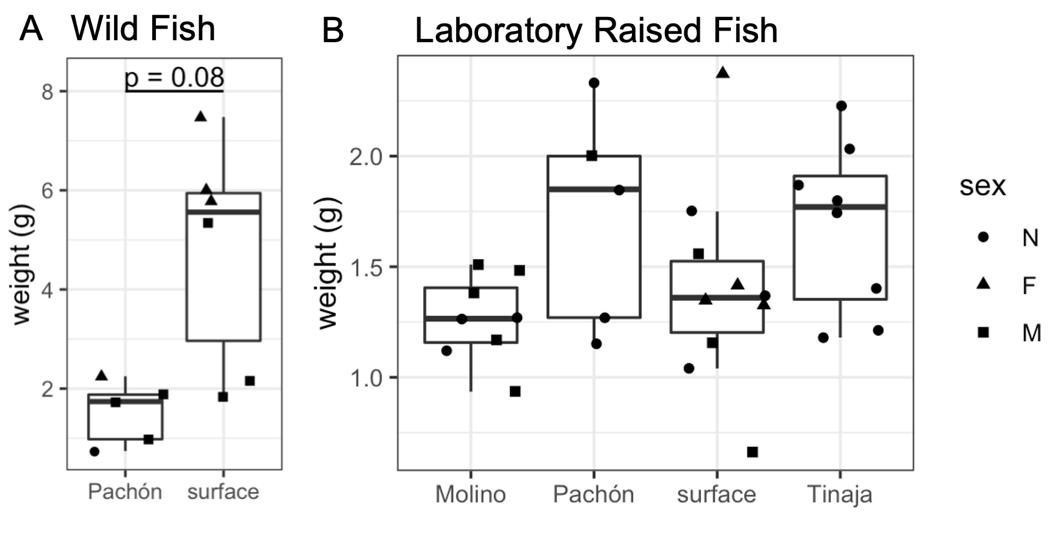
**

**Figure S1.** (A) Weight and sex of wild fish used for intestinal microbiome sequencing. (B) Weight and sex of laboratory raised six-month-old fish used for intestinal microbiome sequencing. Sex determined based on gonad characteristics (N = not differentiated, F = Female, M = Male).


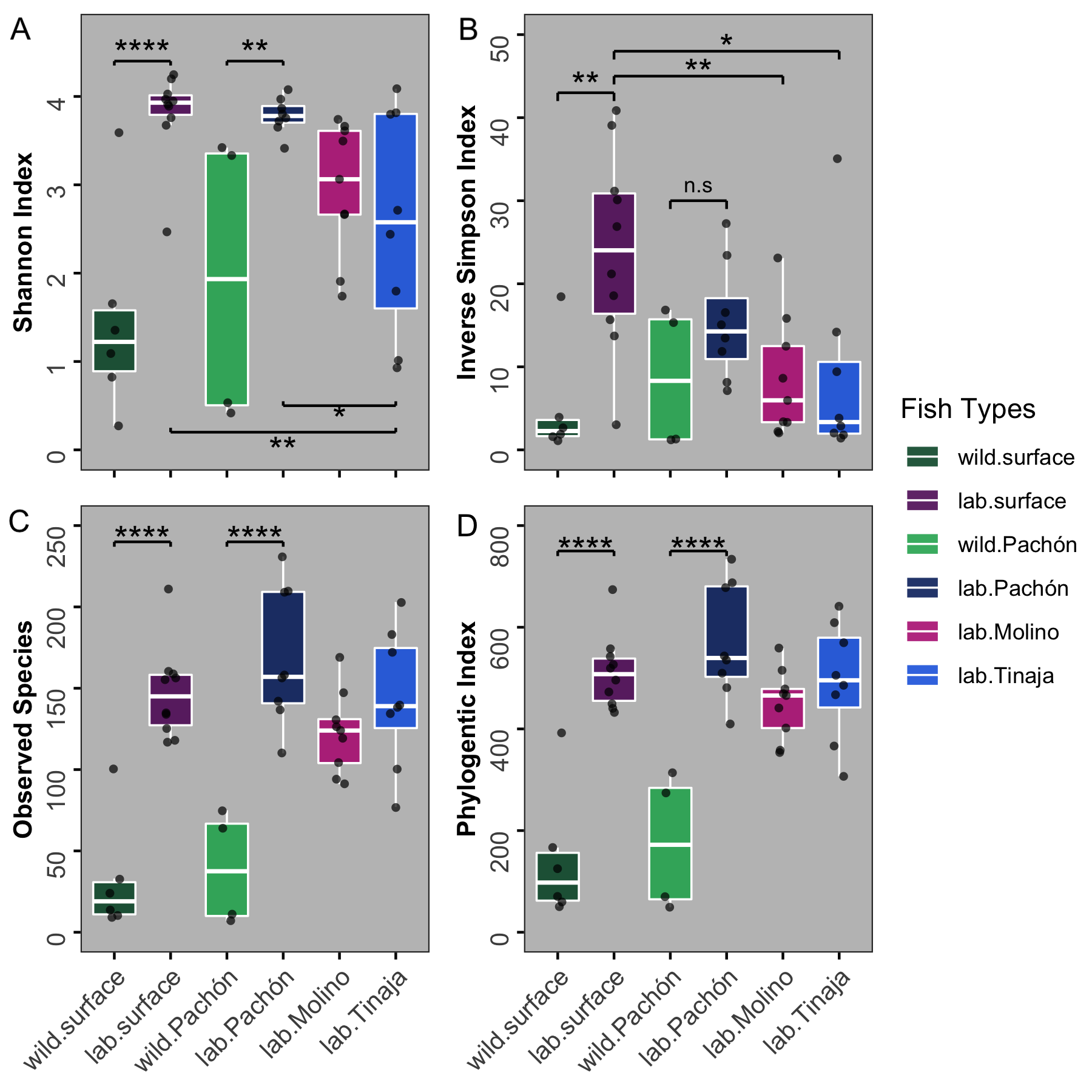


**Figure S2.** Comparison of the number of species and their distributions in intestinal microbiome of *A. mexicanus* surface and cavefish populations as estimated by Shannon Index (A), Inverse Simpson Index (B), Observed Species (C) and Phylogenetic Index (D). Asterisks indicate significance based on one-way ANOVA with Tukey’s post hoc test (*p<0.05, **p<0.005).


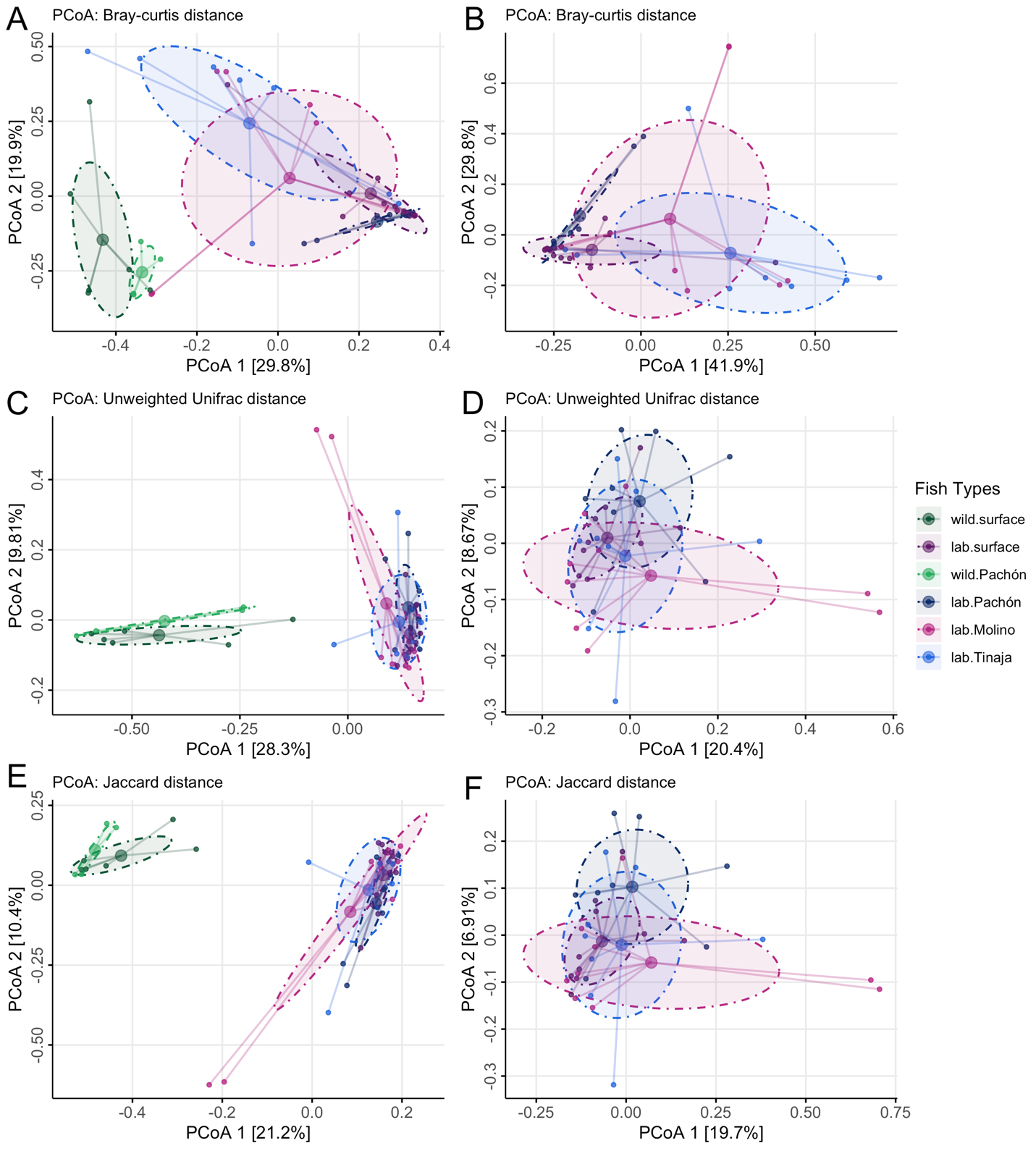


**Figure S3.** Variation in intestinal microbiome composition between *A. mexicanus* surface fish and cavefish in the wild and lab. Distances determined by Bray-Curtis (A, B), Unweighted Unifrac (C, D), and Jaccard (E, F). Ellipses estimate covariance and the centroid of each cluster. PCoA used as the ordination method.

**Figure S4.** Comparison of surface fish and Pachón cavefish core microbiome. (A) Venn diagram showing shared number of ASVs between wild and laboratory-raised *Astyanax mexicanus* from Río Choy River and Pachón cave. (B) Phylogenetic tree of taxa shared between surface fish in the wild and surface fish in the lab. (C) ) Phylogenetic tree of taxa shared between Pachón cavefish in the wild and Pachón cavefish in the lab

**
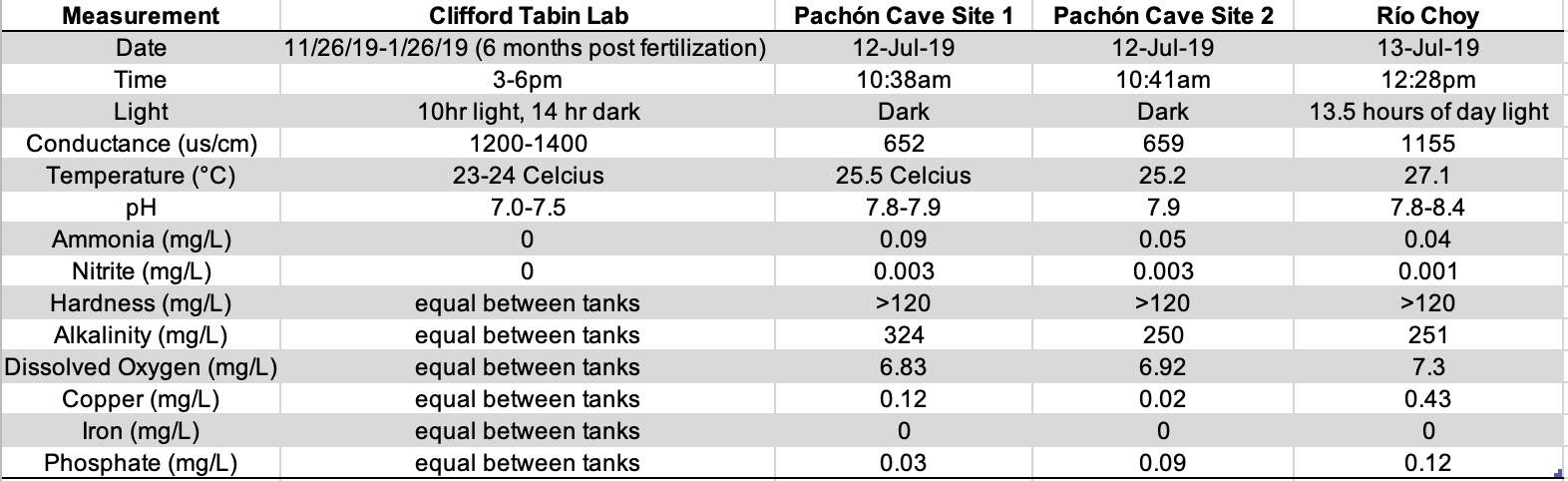
Table S1.** Comparison of environmental variables in wild and laboratory habitats of *A. mexicanus* at the time of fish collection. Data from wild fish collection also reported in Krishnan et al, 2020.(Krishnan et al., 2020)


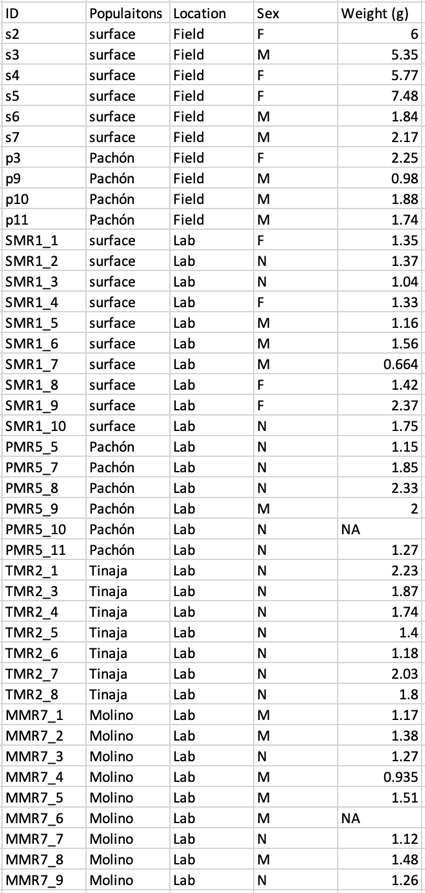


Table S2. Weight and sex of field-collected and laboratory-raised *A. mexicanus* used for 16s rRNA gene sequencing of gut contents.


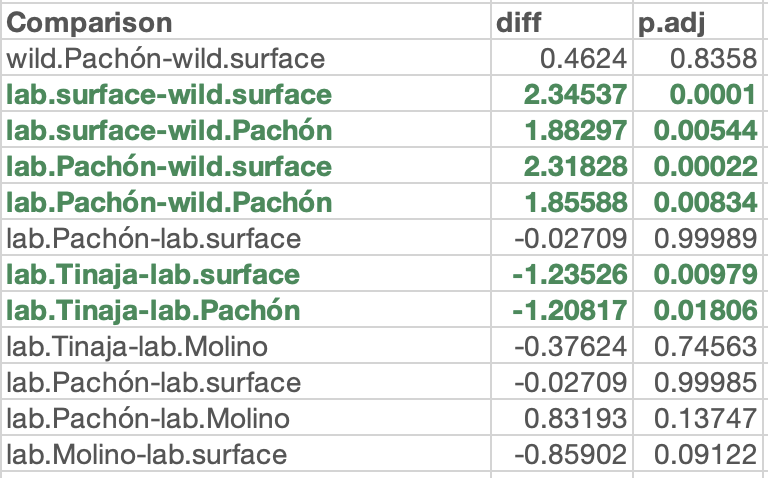


**Table S3.** Statistical comparison of microbiota diversity in *A. mexicanus* gut contents using results of 16s rRNA gene sequencing. Post-hoc pairwise test reveals difference in intestinal microbiome alpha-diversity between *A. mexicanus* morphotypes. FDR method was used to correct P-values for multiple comparisons (p.adjusted). R2 represents the proportion of the variance explained by the independent variable(s).


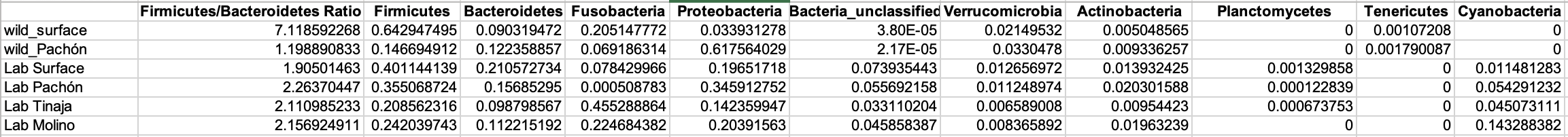


**Table S4**. Firmicutes/Bacteroidetes Ratio and average proportional abundance of bacterial phyla in *A. mexicanus* gut contents.
